# Structural Design, Anticancer Evaluation, and Molecular Docking of Newly Synthesized Ni(II) Complexes with *ONS*-Donor Dithiocarbazate Ligands

**DOI:** 10.3390/molecules29122759

**Published:** 2024-06-10

**Authors:** Claudia C. Gatto, Cássia de Q. O. Cavalcante, Francielle C. Lima, Érica C. M. Nascimento, João B. L. Martins, Brunna L. O. Santana, Ana C. M. Gualberto, Fabio Pittella-Silva

**Affiliations:** 1 University of Brasilia, Institute of Chemistry, Laboratory of Inorganic Synthesis and Crystallography, Brasília 70910-900, DF, Brazil; 2 University of Brasilia, Institute of Chemistry, Laboratory of Computational Chemistry, Brasília 70910-900, DF, Brazil; ericamoreno@unb.br (É.C.M.N.); lopes@unb.br (J.B.L.M.); 3 University of Brasilia, Faculty of Health Sciences and Medicine, Laboratory of Molecular Pathology of Cancer, Brasília 70910-900, DF, Brazil; brunna.los@hotmail.com (B.L.O.S.); pittella@unb.br (F.P.-S.)

**Keywords:** Ni(II) complex, dithiocarbazate, crystal structure, Hirshfeld surface, anticancer activity, molecular docking

## Abstract

The current article reports the investigation of three new Ni(II) complexes with *ONS*-donor dithiocarbazate ligands: [Ni(L^1^)PPh_3_] (**1**), [Ni(L^2^)PPh_3_] (**2**), and [Ni(L^2^)Py] (**3**). Single-crystal X-ray analyses revealed mononuclear complexes with a distorted square planar geometry and the metal centers coordinated with a doubly deprotonated dithiocarbazate ligand and coligand pyridine or triphenylphosphine. The non-covalent interactions were investigated by the Hirshfeld surface and the results revealed that the strongest interactions were π⋅⋅⋅π stacking interactions and non-classical hydrogen bonds C–H···H and C–H···N. Physicochemical and spectroscopic methods indicate the same structures in the solid state and solution. The toxicity effects of the free ligands and Ni(II) complexes were tested on the human breast cancer cell line MCF-7 and non-malignant breast epithelial cell line MCF-10A. The half-maximal inhibitory concentration (IC_50_) values, indicating that the compounds were potent in inhibiting cell growth, were obtained for both cell lines at three distinct time points. While inhibitory effects were evident in both malignant and non-malignant cells, all three complexes demonstrated lower IC_50_ values for malignant breast cell lines than their non-malignant counterparts, suggesting a stronger impact on cancerous cell lines. Furthermore, molecular docking studies were performed showing the complex (**2**) as a promising candidate for further therapeutic exploration.

## 1. Introduction

In recent decades, metal complexes have been extensively studied, especially due to their medicinal chemistry applications [1,2,3,4,5]. They are described as having diverse biological activities; some of them have even been used as chemotherapeutic drugs in different types of cancer treatment [6,7,8,9,10,11]. One of the biggest obstacles with current chemotherapy drugs, such as cisplatin, is their number of adverse effects, including nephrotoxicity, neurotoxicity, nausea, and vomiting [12,13]. This is the motivation behind why many research groups are looking for new compounds that could be alternatives in oncologic treatment with fewer side effects [14,15,16,17,18,19,20].

A class of organic compounds that has been gaining prominence is the dithiocarbazates [17,21,22,23,24]. This type of Schiff base shows a chelating effect and can coordinate with different metal centers by different donor atoms. Furthermore, many dithiocarbazates have already been described in the literature with potential antitumor activity and the complexation of the dithiocarbazate with the metal ions can potentiate their biological action [25,26,27,28,29].

Nickel is present in biological systems and it is possible to find recent research with this metal and dithiocarbazates reported in the literature with biological applications [24,30,31]. Several Ni(II) complexes with dithiocarbazates can interact with the biological environment, showing antitumor, antibacterial, or antifungal potential [15,31]. Previous studies reported a Ni(II) complex derived from S-methyldithiocarbazate showing potent antitumor activity against breast, colon, and liver cancer cell lines, which highlights its IC_50_ and selective index (SI) values [27].

Due to our interest in this class of compounds and their applications, the present study described the synthesis and crystal structures of three new Ni(II) complexes with S-allyl and S-benzyl dithiocarbazates. The metal complexes were characterized by single-crystal X-ray diffraction, FT-IR, UV–Vis, and ^1^H nuclear magnetic resonance. The non-covalent interactions and supramolecular structures were evaluated by Hirshfeld surface analysis. Herein, we report the cytotoxicity of the synthesized compounds against the breast cancer line MCF-7 and healthy breast cell line MCF-10A and their comparison with docking molecular studies. Additionally, molecular docking was carried out to explore the binding affinity of the dithiocarbazate ligands and their Ni(II) complexes with the residues of the active site of trypsin.

## 2. Results and Discussion

**Figure 1:**
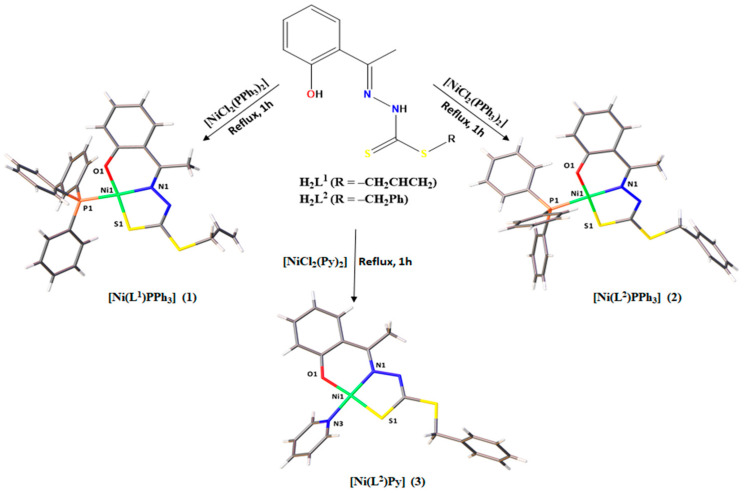
Summary of complexes **(1–3)** obtained from the reactions between dithiocarbazate ligands and Ni(II).

The Ni(II) complexes [Ni(L^1^)PPh_3_] (**1**), [Ni(L^2^)PPh_3_] (**2**), and [Ni(L^2^)Py] (**3**) were obtained by complexation reactions between H_2_L^1^ and H_2_L^2^ ligands and nickel chloride, with triphenylphosphine or pyridine as the coligand to complete the coordination sphere (Figure 1). With the deprotonation of the ligands and the presence of chloride ions, HCl was produced in solution. The crystal structures of complexes (**1–3**) were established by single-crystal X-ray diffraction and all compounds were characterized by spectroscopic and physicochemical methods.

### 2.1. Structural Analyses

**Figure 2:**
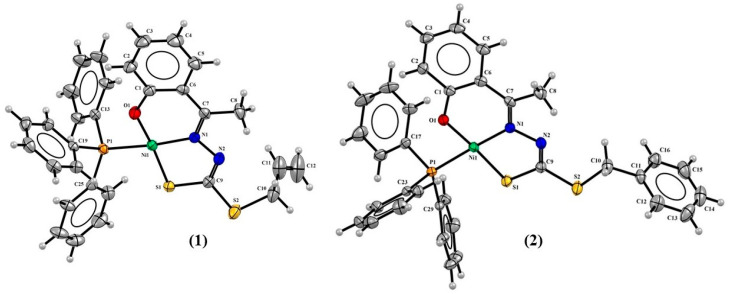
Molecular structures of (**1**) and (**2**) with 30% probability displacement.

**Figure 3:**
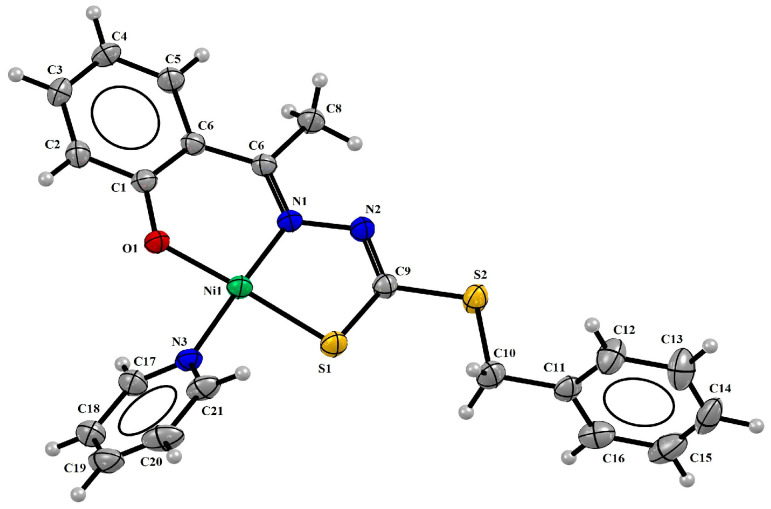
Molecular structure of (**3**) with 30% probability displacement.

The single-crystal X-ray diffraction analyses revealed complexes (**1**) and (**2**) with a square planar geometry, in which the H_2_L^1^ ligand was in a dianionic form and coordinated to the metal center by the O1 from the phenolate function, N1 from the imine, and S2 from the thiolate function (Figure 2). A P1 atom of a triphenylphosphine molecule completed the coordination sphere. The crystal structure of the complex (**3**) also showed a four coordination number for the Ni(II) center, in which the H_2_L^2^ ligand was coordinated in a tridentate way by the ONS donor system, but in this complex, a pyridine molecule completed a planar square environment (Figure 3).

The complexes (**1–3**) showed Ni1-N1 bond lengths between 1.851(3) and 1.896(3) Å, Ni1-S1 lengths between 2.122(12) and 2.133(12) Å, and Ni1-O1 lengths between 1.820(3) Å and 1.836(17) Å. The bond lengths observed with the triphenylphosphine molecule Ni1-P1 were 2.200(8) Å and 2.205(11) Å to (**1**) and (**2**), respectively, and 1.907(3) Å to the pyridine group Ni1-N3. All these distances are very similar to the bond lengths reported in the literature for other Ni(II) complexes [27,32], such as complexes derived from 1,1,1-trifluoro-2,4-pentanedione, whose values of the same coligand chemical bonds were observed between 2.216(3) Å and 2.223(3) Å to Ni1-P1 and 1.915(4) and 1.923(6) Å to Ni1-N1 [15].

The crystal data show the tautomeric change from the thione form in the free ligands to the thiol form in complexes (**1–3**), as evidenced by the N2-C9 bond, whose distances were observed at lower values for the complexes between 1.277(5) Å and 1.295(4) Å compared with distances of 1.353(3) Å and 1.352(3) Å in the free ligands [20,33]. On the other hand, single characteristics were observed for the S1-C9 bond, whose values were calculated between 1.713(4) Å and 1.735(4) Å to the complexes and 1.659(2) Å [20] and 1.661(3) Å to the ligands [20,33]. Table 1 shows selected bond distances and angles for the complexes (**1–3**). 

The square planar geometry proposed for the three complexes is in agreement with the τ_4_ value calculated for each compound [34]. The values were found to be 0.088 for (**1**), 0.045 for (**2**), and 0.072 for (**3**), and are consistent with Ni(II) square planar complexes that have been described [15,18,24,32,35,36]. A small geometry distortion was observed involving the bond angles O1-Ni1-S1 and N1-Ni1-X, where X = P1 to (**1**) and (**2**) and X = N3 to (**3**), whose values varied slightly from 180°. There was also an observed twist in the final part of the ligands for the structures of complexes (**2**) and (**3**), with the twist angle between the planar rings being 90.71(2)° and 75.70(15)°, respectively. 

**Figure 4:**
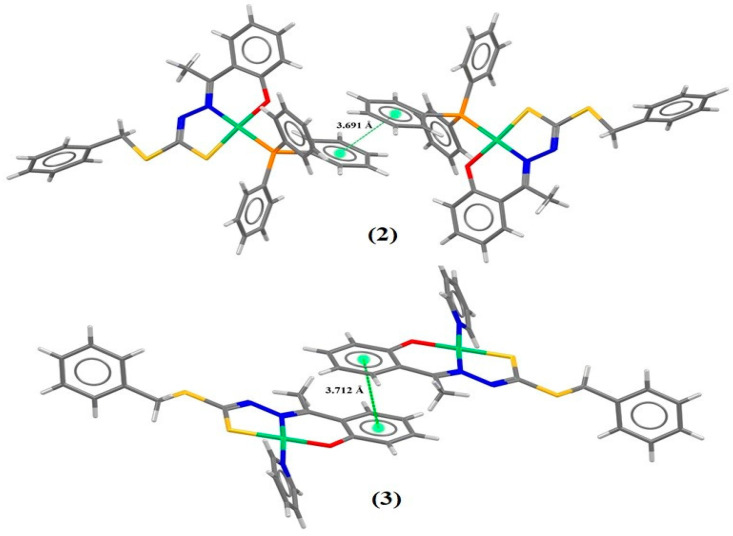
Projection views of (**2**) and (**3**) showing the π⋅⋅⋅π stacking interactions (green dotted lines).

The existence of close phenyl rings along the structural arrangement led to the occurrence of a π⋅⋅⋅π stacking interaction to the (**2**) and (**3**) complexes through the symmetry operator 1 − x, 1 − y, 2 − z, with a displacement of 1.491 Å and distance of 3.691 Å between the centroids of the PPh_3_ rings of complex (**2**), and through the symmetry operator −x, 2 − y, 1 − z, with a shift of 1.160 Å and distance of 3.712 Å between the centroids of the rings of the hydroxyacetophenone of complex (**3**). From these interactions, a unidimensional organization of the asymmetric units along the crystal lattice was possible, corroborating the formation of the crystal structure of the complexes. The π⋅⋅⋅π interactions are represented as depicted in Figure 4.

The crystal data show in complex (**1**) weak non-classical intermolecular hydrogen bonds between C8-H8b⋅⋅⋅N2 of the adjacent molecule with a distance of 2.717(3) Å. In addition, there are observed non-classical intramolecular interactions in C18-H18⋅⋅⋅O1, whose distances were 2.403(3) Å to (**1**) and 2.361(2) Å to (**2**), as illustrated in Figure S1 in the Supplementary Materials. 

### 2.2. Hirshfeld Surface

**Figure 5:**
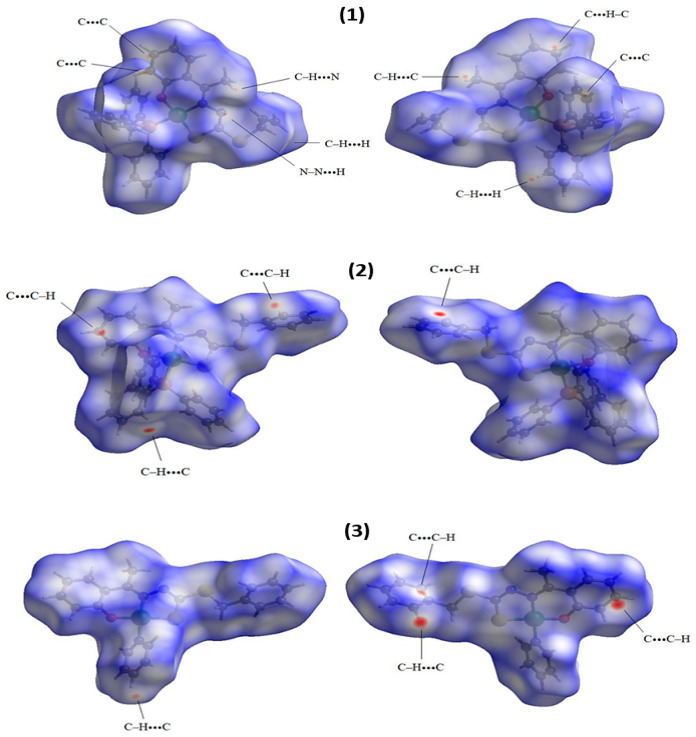
Hirshfeld surfaces of complexes (**1**–**3**) mapped with *d_norm_*.

The Hirshfeld surface (HS) is a complementary tool for structural characterization that allows for a qualitative analysis of the proximity between neighboring molecules and intermolecular interactions. The surface design depends on the interactions associated with the structure, as well as between the atoms of the molecule and the surface properties that promote the contacts, distances, and interactions on the relative strengths of these interactions [15,37]. The first data that can be obtained from the HS mapping by the CrystalExplorer program [38] is the *d_norm_* surface, which shows a pattern of colors ranging from red to blue. The closest contacts between two atoms that are inside and outside the surface are shown as red regions, while the blue color represents the most distant contacts [24,39,40]. Figure 5 shows the 3D *d_norm_* surface mapping for complexes (**1–3**), where the data show that the most common interactions observed were the non-classical hydrogen bonds C–H···H and C–H···N; although in smaller numbers but still present, the interactions π···π were present, with both contacts contributing to the formation of the crystal lattice of the complexes.

**Figure 6:**
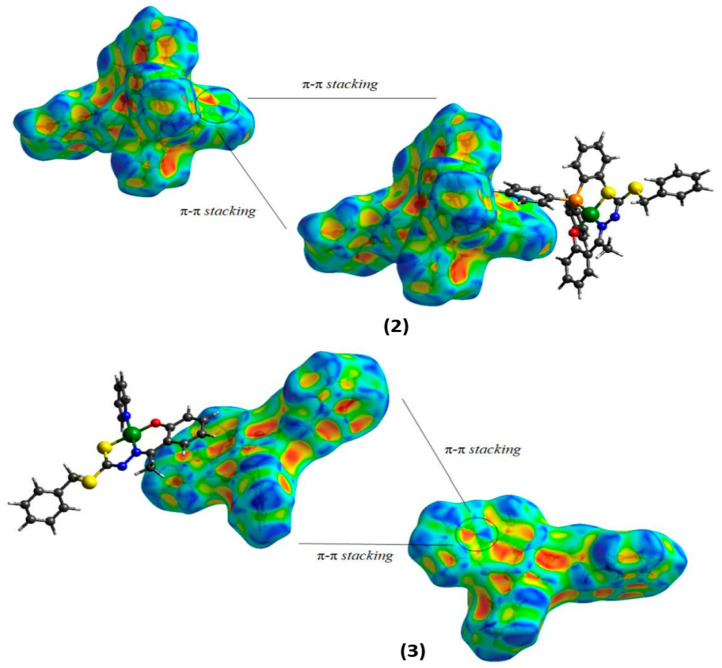
Hirshfeld surfaces mapping shape indexes for (**2**) and (**3**).

The shape index surface, which is also used to evaluate the topology of the surface from intermolecular interactions, is useful to identify interactions of the π···π stacking interactions, where a set of bumps and hollows, such as red and blue triangles on the surface, indicate that π packing occurs between rings of two molecules [15,39,41,42]. The shape index surfaces indicate the presence of π···π interactions between the PPh_3_ rings to complex (**2**), with a distance of 3.691 Å between the centroids, and between dithiocarbazate rings to complex (**3**), with a distance of 3.712 Å between the centroids. These data are from X-ray diffraction and the surfaces are represented in Figure 6.

**Figure 7:**
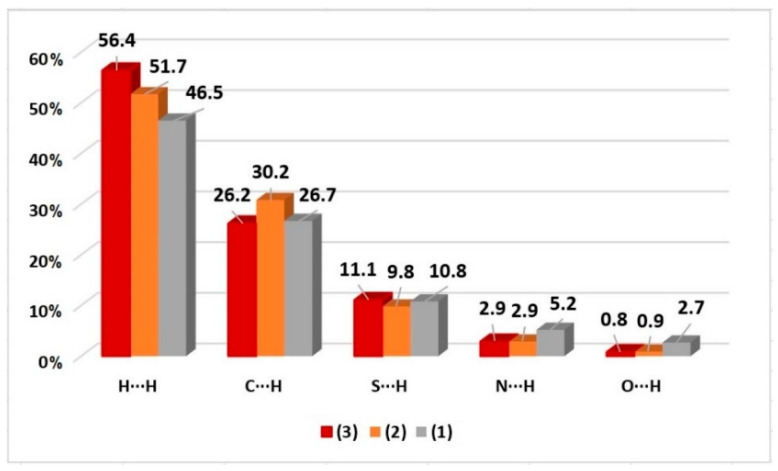
Percentage contributions of the main interactions observed in the fingerprint for complexes (**1–3**).

In addition, fingerprint graphs are also obtained by the CrystalExplorer program [38] and they are used to indicate and quantify different types of intermolecular interactions. The fingerprint plot presents a summary of intermolecular contacts in crystals in a specific range, 0.4–3.0 Å, including reciprocal contacts [39]. Fingerprint graphs were obtained for complexes (**1–3**) and are shown in the Supplementary Materials (Figures S2–S4) and summarized in Figure 7. The results indicate that the greatest contributions of the interactions were for H···H, C···H, S···H, N···H, and O···H for the compounds studied, with contacts contributing between 0.8% and 56.4%. 

### 2.3. Infrared Spectra

The FT-IR spectra obtained for the complexes and ligands are shown in the Supplementary Materials (Figures S5–S9). The main bands observed are shown in Table 2. Comparing the spectra of the free ligands and complexes, the disappearance of two bands after complexation could be attributed to ν(O-H) and ν(N-H) stretching. These data are in agreement with the proposed structures since there was the deprotonation of the N1 and O1 atoms and subsequent coordination of the ligand to the Ni(II) center in a doubly deprotonated way. 

Moreover, other evidence of Ni-N coordination was the band observed in around 1601 cm^−1^, which was attributed to ν(C=N) stretching of azomethine in the free ligands spectra, which decreased to lower wavenumbers after complexation, with a variation of 40–79 cm^−1^ observed [20,43,44,45]. An increase in the ν(C=N) bond length was also observed by single-crystal X-ray diffraction, with lengths in the range of 1.293–1.297 Å for the free dithiocarbazates and 1.313–1.330 Å for the complexes. 

The Ni-S coordination could be observed by the decreased frequency of the band corresponding to the ν(CSS) asymmetric stretching that varied from the range of 1057–1101 cm^−1^ in the free ligands to 939–984 cm^−1^ in the complexes. This behavior was already observed in similar structures and indicates a change in tautomerism (thione to thiol) after the coordination to the metal center [15,21,46].

Concerning the complexes formation, characteristic bands of the ν(C_PPh3_-C_PPh3_) triphenylphosphine stretching were observed at 1435 cm^−1^ (**1**) and 1434 cm^−1^ (**2**) and bands were also detected at 1070 cm^−1^ (**1**) and 1094 cm^−1^ (**2**), which referred to ν(C_PPh3_-P_PPh3_) stretching [15,32,47,48]. Bands between 692 and 693 cm^−1^ were observed, which were already attributed in other studies as ν(Ni-P_PPh3_) stretching [15,27,32,49]. On the other hand, the FT-IR spectrum for complex (**3**) also showed two characteristics bands that referred to ν(C=N)_py_ and δ(Py) from the pyridine coligand in the same range as other reported complexes [15,28,32,50].

### 2.4. Electronic Spectra

The electronic spectra of ligands and complexes (**1–3**) are shown in the Supplementary Materials (Figures S10 and S11 and Table S1). The absorption spectra of all compounds showed bands in the range of 292–308 nm that could be attributed to the electronic transition π→π* of the azomethine function; furthermore, bands between 361–383 nm were observed that could be related to the n→π* of dithiocarbazate moiety [15,27,48]. On the other hand, when comparing the spectra of ligands and complexes, bathochromic and hypochromic effects were observed. The shifts in bands to a lower energy are evidence of complexation [18,27,32].

In addition, the spectra of the (**1–3**) complexes showed bands at 428 nm that could be attributed to the ligand–metal charge transition (LMCT), as justified by their high values of molar absorptivity and wide spectral range, which corresponded to a S→Ni(II) transition, as indicated in other studies [18,27,32]. The d-d transition bands expected in the regions 385–420 nm were obscured by the LMCT bands and were not observed in the spectra of the Ni(II) complexes [27,51].

### 2.5. ^1^H NMR Spectra

The ^1^H NMR and ^13^C NMR spectra for the ligands and complexes (**1–3**) are found in the Supplementary Materials (Figures S12–S21). The compounds were not completely soluble in water; due to this solubility, the spectra were measured in DMSO-d_6_. The data observed in the ^1^H NMR spectra of (**1–3**) complexes show shielded signals appearing as singlets in the ranges of 2.50–2.85 ppm and 3.69–5.50 ppm, which could be attributed to the hydrogens H5 and H6 of the -CH_3_ groups and -CH_2_, respectively. Furthermore, in the spectra of all complexes, hydrogens H1 to H4 could be observed in a range characteristic of aromatic hydrogens [20,22].

The signs of the allyl substituent in the (**1**) spectrum are presented in a very characteristic way. The multiplet in 5.85 ppm was assigned to the hydrogen -CH=, and the doublets in 5.03 ppm and 5.17 ppm were designated to the terminal hydrogens (=CH_2_), which were in the chemical shifts of cis and trans hydrogens, respectively [29]. On the other hand, the aromatic hydrogens of the aryl substituent in the (**2**) and (**3**) spectra could be assigned to the signs in the range of 7.31–7.78 ppm [21,32,52,53].

In addition, in the spectra of complexes (**1**) and (**2**), signals corresponding to the number of hydrogens in the PPh_3_ group could be observed in the expected range of 7.34–7.74 ppm; similarly, signals were observed in the most unshielded range of the spectrum of complex (**3**), which could be attributed to the hydrogens of the pyridine coligand [15,27,32]. Further evidence of complexation was the absence of the NH band in the spectra of the three complexes, which suggests deprotonation of the ligand during complexation and coordination through the thiolate form, as also suggested by other analyses that have already been presented.

The ^13^C NMR spectra of the complexes (**1–3**) show signals corresponding to -CH_3_ and -CH_2_ groups in the ranges of 17.94–19.04 ppm and 37.76–39.15 ppm, respectively. Additionally, signals corresponding to the carbons of the aromatic rings were observed between 116.15 and 152.04 ppm. Furthermore, the spectra of the complexes show signs that could be attributed to the carbons of the Py and PPh_3_ coligands. Finally, the C9 carbon signal also helped to confirm that the ligands were coordinated to the metal atom by the sulfur atom, as these signals appeared at lower values (164.31–169.9 ppm) compared with the free ligands, in which this signal appeared in the range of 195.97–196.08 ppm.

### 2.6. Biological Activity Analysis

The in vitro cytotoxicities of the ligands H_2_L^1^ and H_2_L^2^ and the complexes (**1–3**) were evaluated against the human breast cancer cell line MCF-7 and the non-malignant breast epithelial cell line MCF-10A. Related work shows that NiCl_2_⋅6H_2_O had no substantial effect on cell proliferation or cell viability in the cells tested [54]. The measurement was performed using the MTT assay in cultures treated with increasing concentrations. All compounds showed cytotoxic potential and suppressed the proliferation of MCF-7 cells. The proliferative ability of MCF-7 cells of all tested compounds at 24 h, 48 h, and 72 h was significantly reduced with increasing concentrations (Table 3). 

**Figure 8:**
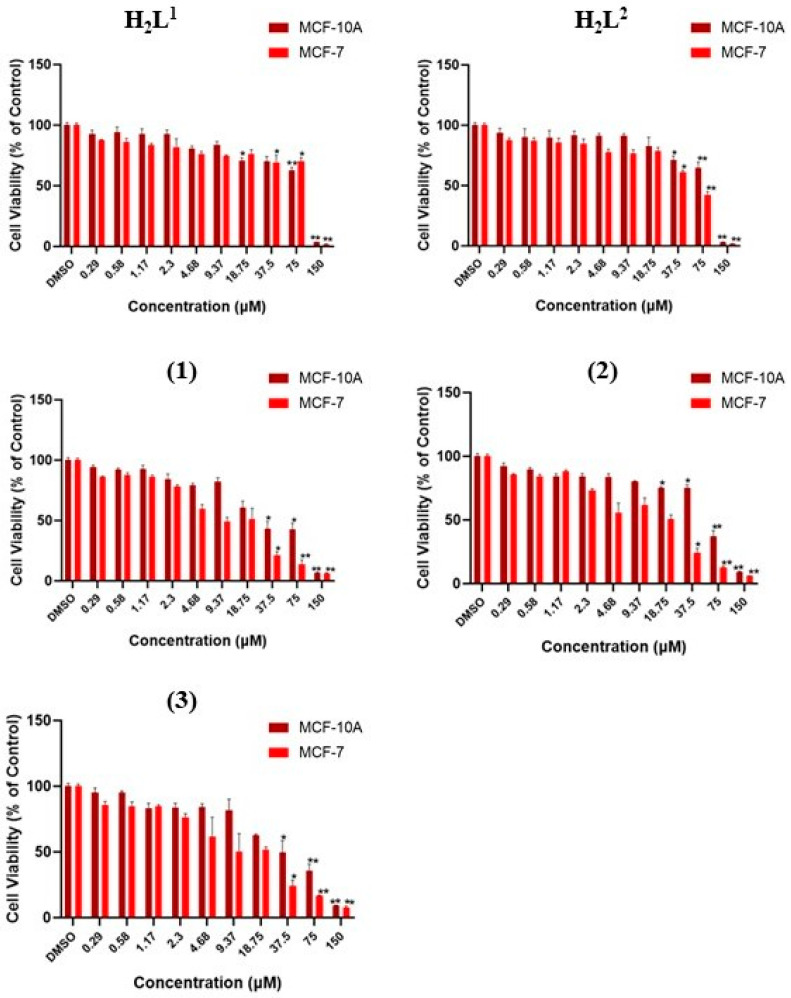
Evaluation of cytotoxic effects by MTT assay at 72 h. DMSO at 0.01% did not affect the cell viability of cell lines. The asterisk indicates that cell viability was significantly different from the respective DMSO control (* *p* < 0.5, ** *p* < 0.001 Kruskal–Wallis followed by Dunn’s comparison test).

The data show that the dithiocarbazate ligands were active against MCF-7 cells, emphasizing the results after 72 h of exposure (Figure 8, Figures S22 and S23). It is possible to observe that H_2_L^2^ presented the best results, with an IC_50_ of 23.97 µM compared with 28.49 µM presented by H_2_L^1^. Furthermore, H_2_L^2^ presented higher selectivity when compared with non-malignant MCF-10A cells, presenting higher selectivity indexes at 24 h, 48 h, and 72 h than H_2_L^1^ [27]. These results indicate that variations in the -R groups of the dithiocarbazate structures could modify the observed activities, and the ligand with the -CH_2_Ph group showed better activity than the -CH_2_CHCH_2_ group. Other studies with different cell lines also found greater cytotoxicity with changes in steric properties of the ligands and lipophilicity [29,55,56,57].

**Figure 9:**
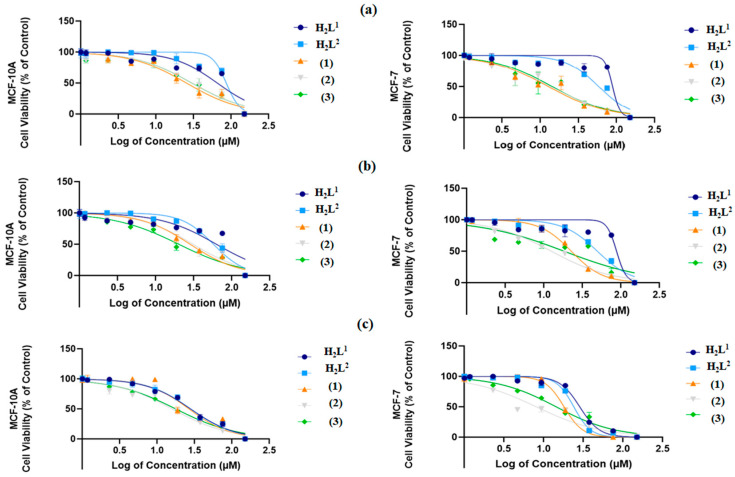
Concentration–response curves of the tested compounds in both MDA-MB-231 and MCF-7 cells after 24 h exposure in (**a**), 48 h exposure in (**b**), and 72 h exposure in (**c**). Concentrations are shown as logarithms to perform the non-linear data regression, allowing the concentration calculation to inhibit 50% of cell viability in cultures (IC_50_).

On the other hand, all complexes showed significant cytotoxicities at 37.5 µM, 75 µM, and 150 µM, with dose-dependent toxicities in both cell lines with an expressive effect at 72 h. In addition, analysis of the dithiocarbazate H_2_L^1^ and H_2_L^2^ and their complexes (**1–3**) at various concentrations in tumor and non-tumor lines allowed us to plot the concentration–response curves of all tested compounds (Figure 9). 

In general, the results show that the complexes (**1–3**) were stronger than their respective ligands, which indicates that the complexation with the Ni(II) center enhanced the observed activity. For comparison purposes, at 72 h, the IC_50_ values were calculated to be between 23.97 and 28.49 µM for the free ligands and between 8.01 and 18.07 µM for the complexes. Moreover, the complexes also showed higher selectivity than the ligands, which indicates that they are potential drugs; this is clear given their calculated SI values (Table 4).

Evaluating the structure–activity relationship, a higher activity of complex (**2**) was observed compared with complex (**1**); this was the same behavior shown by their respective ligands, with the S-benzyl substituent having a lower IC_50_ than the S-allyl group. On the other hand, when we compared complex (**2**) and complex (**3**), where both were derived from the H_2_L^2^ ligand, complex (**2**), which had the triphenylphosphine ligand, presented better results than complex (**1**), which had pyridine as the ligand. These two comments suggest that the increase in phenyl groups in the molecule enhanced the activity. A similar behavior was observed in a recent study with the MCF-7 cell line, in which a Cu(II) complex derived from a S-benzyl-dithiocarbazate ligand presented a significantly better IC_50_ value (11 µM) than its analog derived from S-methyl-dithiocarbazate (46 µM) [58].

Notably, complex (**2**) showed the most promising anticancer potential among all compounds tested, with an IC_50_ value (8.073 ± 0.011 µM, 72 h) lower than that already reported for common reference compounds, such as cisplatin (38.24 µM, 72 h) [59] and tamoxifen (11.20 µM, 72 h) [25], in a similar MTT assay. In addition, complex (**2**) presented the best selectivity for malignant breast cell lines compared with their non-malignant counterpart, in which SI = 3.85 at 72 h of exposure. Studies have already suggested that compounds that have SI ≥ 3 are considered highly selective for a specific cell line [27,60].

### 2.7. Molecular Docking

The molecular docking study revealed a good agreement with the experimental biological activity assay performed with the dithiocarbazate ligands and complexes. These molecules showed similarity in the inhibitory interaction profile as the Ptry9 peptide derivative from a Bowman–Birk inhibitor (BBI) [61]. As is known, the BBIs molecules present carcinogenesis suppressor action in a huge variety of cancers in animal cells, as well as anti-inflammatory action in the inflammation process [62,63,64].

As shown in Table 4, complex (**2**) presents the best-predicted energy of binding (−9.20 kcal/mol) in the inhibitory process against the trypsin enzyme, followed by complexes (**2**) and (**1**), respectively. The ligand H_2_L^2^ presented the best result (−5.80 kcal/mol) when compared with the H_2_L^1^ ligand, showing the lowest value of the binding energy in the docking study.

In comparison with the results of the biological assay in the MCF-7 cells, after 72 h of exposure, shown previously in Table 3, the same trend of the classification in the inhibitory activity was observed in the in silico prediction performed with the AutoDock Vina algorithm, which performed a Ptry9L cytotoxicity experimental assay against the MDA.MB.231 breast cancer cell type and the non-malignant breast epithelial cell line MCF-10A, which are the same normal mammary epithelial cells used in the present study [61]. The authors applied the same protocol used in this study, where the measurement was performed using an MTT assay [61].

According to the molecular modeling study, the dithiocarbazate compounds that presented the -CH_2_Ph group in their molecular structure expressed more activity than the compounds with the -CH_2_CHCH_2_ group in their molecular composition. In general, the results showed the main function of the complexation with the Ni(II) atom when comparing the inhibitory binding energy of the complexes and their ligands separately. The complexes showed scores higher than −6.50 kcal/mol, while the ligands presented scores under −6.00 kcal/mol. 

Comparing complexes (**1**) and (**2**), both presented the triphenylphosphine group, which permitted the formation of a hydrogen bond between the S2 atom and the NE2:Gly192 main residue of the oxyanion hole of the active site of the trypsin enzyme. The combination of the presence of the triphenylphosphine group and the structure of the complex with S-benzyl dithiocarbazate indicate the best molecular arrangement to inhibit the trypsin enzyme, as well as the cancer cell MCF-7 type.

**Figure 10:**
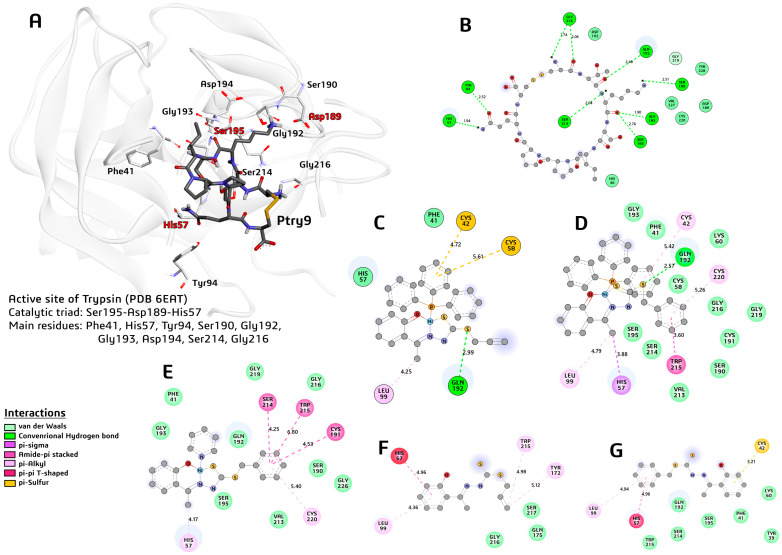
A 3D view of the trypsin–Ptry9L complex and 2D views of trypsin–compound complexes. (**A**) Main residues of the active site of the enzyme, and the Ptry9L (represented in tube and dark color). (**B**) A 2D representation of the main interactions and respective distance of the trypsin and Ptry9L. (**C**) A 2D representation of the main interactions and respective distance of the active site of the trypsin and complex (1). (**D**) A 2D representation of the main interactions and respective distance of the trypsin and complex (2). (**E**) A 2D representation of the main interactions and respective distance of the trypsin and complex (3). (**F**) A 2D representation of the main interactions and respective distance of the trypsin and H_2_L^1^ ligand. (**G**) A 2D representation of the main interactions and respective distance of the trypsin and H_2_L^2^ ligand.

The interaction profile is shown in Figure 10, which shows the correlation between the number of interactions made with the main residues of the active site of the trypsin and the compounds studied here. In all cases, the higher affinity indicated by the energy of binding (score) was observed in the compounds that formed a large number of interactions with the catalytic triad residues (Ser195-Asp189-His57) and additionally with the oxyanion hole residues, such as the Gly192, Gly193, and Gly216 residues, mainly as non-covalent interactions, such as the hydrogen bond type, pi-sigma, amide-pi stacked, and long-range van der Waals, at a distance of 6.0 Å.

The best inhibitor was the molecule Ptry9L, which showed an energy of binding of −10.70 kcal/mol. This peptide derivative of the BBI type shows high affinity with the active site residues, forming nine hydrogen bonds with distances between 1.74 and 2.76 Å. The interaction between the Lis3:O(Ptry9L)….N:Ser195(Trypsin) atoms at 2.76 Å is highlighted in Figure 10B. On the other hand, none of the other compounds studied performed strong interactions with the Ser195 catalytic residue.

Considering the complexes and ligands proposed in this study, complex (**2**) was the compound best able to inhibit trypsin. This molecule formed a strong hydrogen bond between the S2:(complex **2**)….NE2:Gly192(trypsin) atoms around 2.57 Å, as well as pi-sigma interactions between its C8:(complex **2**)….His57(centroid) at 3.88 Å. Complex (**2**) performed a van der Waals interaction type with a lot of residues of the active site as the Phe41, Gly193, Gly216, Gly219, Ser 195, Ser214, and Ser190. The complex (**2**) mimicked part of the Ptry9L interactions profile and presented the best score in the inhibitory action against trypsin.

## 3. Materials and Methods

### 3.1. Materials, Methods, and Instruments

The reagents and solvents used in this work for all syntheses and characterizations were obtained from a commercial source (Merck, Boston, MA, USA) without additional purification. The determination of the CHN mass percentages of the synthesized compounds was performed through elemental analysis carried out in the Perkin Elmer/Series II 2400 analyzer (Shelton, CT, USA). The vibrational spectra (FT-IR) were obtained using the Varian 640 spectrophotometer (Agilent, Santa Clara, CA, USA), being defined as the scan in the region of 4000–400 cm^−1^, with a resolution of 4 cm^−1^, and each spectrum was obtained with 16 acquisitions. The samples were analyzed in a solid pellet form (1.0 mg of the compound/100.0 mg of KBr). Molecular absorption spectra in the ultraviolet–visible region (UV–Vis) were obtained using an Agilent HP 8453 spectrophotometer (Santa Clara, CA, USA), and the spectra were determined using solutions with concentrations of 2 × 10^−5^ mol·L^−1^ in dimethylformamide (DMF). ^1^H nuclear magnetic resonance spectra were obtained on a BRUKER Avance III HD 14T spectrometer (Billerica, MA, USA), and the samples were prepared with deuterated dimethylsulfoxide solvent (DMSO-d_6_) and tetramethylsilane as an internal reference. 

### 3.2. Synthesis of S-Allyl-2-(2-hydroxyphenyl-ethylidene)dithiocarbazato (H_2_L^1^)

The synthesis was followed according to a related methodology [20]. Yield: 149 mg (70%). Melting point: 116 °C. Elemental analyses calculated for C_12_H_14_N_2_S_2_O: C, 54.11; H, 5.28; N, 10.52. Found: C, 53.86; H, 5.14; N, 10.65. ^1^H NMR (DMSO-d_6_ δ, ppm): 2.47 (s, 3H, CH_3_); 3.90 (d, *^3^J* = 7.00 Hz, 2H, S-CH_2_); allyl groups: 5.18 (d, *^3^J_cis_* = 9.90 Hz, H, CH=CH_cis_), 5.35 (d, *^3^J_trans_* = 16.87 Hz, H, CH=CH*_trans_*,), 5.93 (m, H, -CH=); aromatic rings: 7.64 (d, *^3^J_orto_* = 7.70 Hz, 1H, -CH^2^_Ar_=), 7.36 (t, *^3^J_orto_* = 7.70 Hz, H, -CH^3^_Ar_=), 6.94 (m, 2H, -CH^4^_Ar_= and -CH^5^_Ar_=), 11.40 (s, H, N-H). ^13^C NMR (DMSO-d_6_ δ, ppm): 15.67 (CH_3_), 36.44 (S-CH_2_); allyl groups: 117.21 and 129.44; aromatic rings: 117.21–132.62, 157.98 (C=N), 195.97 (C=S). FT-IR spectra selected bands (ν/cm^−1^): ν(O-H) 3245, ν(N-H) 3114, ν(CSS) 1101, ν(C=N) 1601, ν(C-O) 1219, ν(N-N) 936. UV-Vis (DMF): λ_max_ = 294 nm and 361 nm.

### 3.3. Synthesis of S-Benzyl-2-(2-hydroxyphenyl-ethylidene)dithiocarbazato (H_2_L^2^)

The synthesis was followed according to a related methodology [20]. Yield: 268 mg (85%). Melting point: 145 °C. Elemental analyses calculated for C_16_H_16_N_2_S_2_O: C, 52.01; H, 4.07; N, 16.17. Found: C, 51.58; H, 3.66; N, 15.77. ^1^H NMR (DMSO-d_6_ δ, ppm): 2.47 (s, 3H, CH_3_), 4.55 (s, 2H, S-CH_2_); aromatic rings: 6.90 (m, 2H, -CH^4^_Ar_= and -CH^5^_Ar_=), 7.27 (t, *^3^J_orto_* = 7.43 Hz, H, -CH^11^_Ar_=), 7.32 (m, 2H, -CH^9^_Ar_= and -CH^13^_Ar_=), 7.33 (m, H, -CH^3^_Ar_=), 7.43 (d, *^3^J_orto_* = 7.78 Hz, 2H, -CH^10^_Ar_= and -CH^12^_Ar_=), 7.70 (d, *^3^J_orto_* = 7.86 Hz, H, –CH^2^_Ar_=), 11.30 (s, H, O-H), 12.75 (s, H, N-H). ^13^C NMR (DMSO-d_6_ δ, ppm): 15.81 (CH_3_), 37.98 (S-CH_2_); aromatic rings: 116.91–136.31, 157.38 (C-O), 157.91 (C=N), 196.08 (C=S). FT-IR spectra selected bands (ν/cm^−1^): ν(O-H) 3418, ν(N-H) 3179, ν(CSS) 1057, ν(C=N) 1601, ν(C-O) 1228, ν(N-N) 987. UV-Vis (DMF): λ_max_ = 292 nm and 376 nm.

### 3.4. Synthesis of [Ni(L^1^)PPh_3_] *(**1**)*

A total of 0.2 mmol (52.4 mg) of triphenylphosphine (PPh_3_) previously dissolved in 5 mL of MeOH and 0.1 mmol (23.7 mg) of NiCl_2_⋅6H_2_O in 5 mL of MeOH were refluxed for 1 h. After this time, 0.1 mmol (26.6 mg) of H_2_L^1^ dissolved in 5 mL of methanol was added, and the reaction continued for another 1 h at reflux. Red crystals suitable for X-ray diffraction were obtained after slow evaporation of the solvent. Yield: 32.0 mg (56%). Melting point: 163 °C. Elemental analysis for C_30_H_27_NiN_2_OPS_2_ calcd: C, 62.12; H, 4.92; N, 5.06. Found: C, 61.79; H, 4.62; N, 5.34. ^1^H NMR (DMSO-d_6_ δ, ppm): 2.77 (s, 3H, CH_3_), 3.69 (d, *^3^J* = 7.00 Hz, 2H, S-CH_2_); allyl groups: 5.03 (d, *^3^J_cis_* = 9.90 Hz, H, CH=CH*_cis_*), 5.17 (d, *^3^J_trans_* = 16.90 Hz H, CH=CH*_trans_*), 5.85 (m, H, -CH=); aromatic rings: 6.19 (d, *^3^J_orto_* = 7.70 Hz H, –CH^1^_Ar_=), 6.59 (t, *^3^J_orto_* = 7.70 Hz, H, -CH^2^_Ar_=), 7.02 (t, *^3^J_orto_* = 7.70 Hz H, -CH^3^_Ar_=), 7.52 (m, 2H, -CH^4^_Ar_= and PPh_3_). ^13^C NMR (DMSO-d_6_ δ, ppm): 17.94 (CH_3_), 37.76 (S-CH_2_); allyl groups: 116.21 and 130.88; aromatic rings: 118.59–132.36, 164.27 (C=N), 169.96 (C=S); PPh_3_: 129.12–134.47. FT-IR spectra selected bands (ν/cm^−1^): ν(CSS) 982; ν(C=N) 1523; ν(C-O) 1239; ν(N-N) 1023; ν(PPh_3_) 1070, 1435, and 692. UV-Vis (DMF): λ_max_ = 304 nm, 379 nm, and 428 nm.

### 3.5. Synthesis of [Ni(L^2^)PPh_3_] *(**2**)*

A total of 0.2 mmol (52.4 mg) of triphenylphosphine (PPh_3_) previously dissolved in 5 mL of MeOH and 0.1 mmol (23.7 mg) of NiCl_2_⋅6H_2_O in 5 mL of MeOH were refluxed for 1 h. After this time, 0.1 mmol (31.6 mg) of H_2_L^2^ dissolved in 5 mL of methanol was added, and the reaction continued for another 1 h at reflux. Red crystals suitable for X-ray diffraction were obtained after slow evaporation of the solvent. Yield: 36.9 mg (59%). Melting point: 197 °C. Elemental analysis for C_34_H_29_NiN_2_OPS_2_ calcd: C, 67.68; H, 4.85; N, 4.64. Found: C, 67.91; H, 4.26; N, 4.46. ^1^H NMR (DMSO-d_6_ δ, ppm): 2.85 (s, 3H, CH_3_), 4.35 (s, 2H, S-CH_2_); aromatic rings: 6.25 (d, *^3^J_orto_* = 7.70 Hz, H, -CH^1^_Ar_=), 6.64 (t, *^3^J_orto_* = 7.70 Hz, H, -CH^2^_Ar_=), 7.09 (m, 2H, -CH^3^_Ar_= and -CH^4^_Ar_=), 7.31–7.78 (-CH^7−11^_Ar_= and PPh_3_). ^13^C NMR (DMSO-d_6_ δ, ppm): 18.03 (CH_3_), 39.09 (S–CH_2_); aromatic rings: 116.24–138.02, 161.43 (C=N), 164.31 (C=S); PPh_3_: 129.16–134.41. FT-IR spectra selected bands (ν/cm^−1^): ν(CSS) 939; ν(C=N) 1561; ν(C-O) 1156; ν(N-N) 1014; ν(PPh_3_) 1094, 1434, and 693. UV-Vis (DMF): λ_max_ = 308 nm, 383 nm, and 428 nm.

### 3.6. Synthesis of [Ni(L^2^)Py] *(**3**)*

A total of 0.1 mmol (8 mg) of pyridine (Py) and 0.1 mmol (23.7 mg) of NiCl_2_⋅6H_2_O in 5 mL of MeOH were refluxed for 1 h. After this time, 0.1 mmol (31.6 mg) of H_2_L^2^ dissolved in 5 mL of methanol was added, and the reaction continued for another 1 h at reflux. Red crystals suitable for X-ray diffraction were obtained after slow evaporation of the solvent. Yield: 28.1 mg (62%). Melting point: 149 °C. Elemental analysis for C_21_H_19_NiN_3_OS_2_ calcd: C, 58.62; H, 6.79; N, 9.77. Found: C, 59.06; H, 5.96; N, 9.22. ^1^H NMR (DMSO-d_6_ δ, ppm): 2.50 (s, 3H, CH_3_), 5.50 (s, 2H, S–CH_2_); aromatic rings: 6.52 (m, 2H, -CH^1^_Ar_= and CH^4^_Ar_=), 6.87 (m, 2H, -CH^2^_Ar_= and CH^3^_Ar_=), 7.63 (m, 5H, -CH^7−11^_Ar_=); pyridine rings: 7.28 (m, 2H, –CH^13^_Ar_= and -CH^15^_Ar_=), 7.65 (m, 2H, -CH^12^_Ar_= and -CH^16^_Ar_=), 7.28 (m, H, -CH^14^_Ar_=). ^13^C NMR (DMSO-d_6_ δ, ppm): 19.04 (CH_3_), 39.15 (S–CH_2_); aromatic rings: 116.15–152.04, 161.52 (C=N), 169.31 (C=S); Py: 128.93–137.87. FT-IR spectra selected bands (ν/cm^−1^): ν(CSS) 984, ν(C=N) 1522, ν(C-O) 1239, ν(N-N) 1070, δ(Py) 690, ν(C=N)_py_ 1603. UV-Vis (DMF): λ_max_ = 305 nm, 381 nm, and 428 nm.

### 3.7. Crystal Structure Determination

Data collection of the compounds was undertaken by single-crystal X-ray diffraction and performed on a Bruker CCD SMART APEX II diffractometer in which a graphite monochromator (Bruker-AXS, Karlsruhe, Germany) was used that had a Mo-Kα (0.71073 Å) source at 293 K. The structures were solved using SHELXS-2018/3 [65] and the refinement was accomplished using SHELXL-2018/3 [66] with a minimization of least squares. The determination of the positions of the non-hydrogen atoms was carried out using successive Fourier differences and the refinement was carried out with anisotropic parameters using the OLEX2 program [67]. Molecular graphics were generated via MERCURY 2023.2.0 software [68]. The experimental details and refinement results are summarized in Table S2. CCDC nos. 2304153 for (**1**), 2304154 for (**2**), and 2304155 for (**3**) contain supplementary crystallographic data.

### 3.8. Computational Details

The Hirshfeld surfaces (HSs) were obtained using the CrystalExplorer 21.5 program [38], in which crystallographic information files (CIFs) obtained from experimental measurements of single-crystal X-rays were used as input files. The first data obtained were the 3D *d_norm_* surfaces (normalized contact distance), which were mapped in a fixed red (−0.2000 Å) and blue (1.4000 Å) color scale for all molecules. According to the *d_norm_* surface, it is possible to visualize the regions involved in contacts between donor and recipient regions. A second set of data obtained was used to map the surface in the shape index function, which was used to evaluate the topology of the surface from the intermolecular interactions, and it was very useful in the identification of π···π stacking interactions. In addition, fingerprint plots were obtained for the three complexes to quantitatively evaluate all the contacts involved in the formation of the crystalline network of the compounds.

### 3.9. Biological Activity

The effect of the compounds applied to breast cancer MCF-7 cells and breast epithelial MCF-10A cells was evaluated by measuring 3-(4,5-dimethylthiazol-2-yl)-2,5-diphenyl tetrazolium bromide (MTT) (Invitrogen, Waltham, MA, USA) [69]. All compounds were diluted in dimethylsulfoxide (DMSO) with a final DMSO concentration of 0.01% (0.01 mL/100 mL × 100 = 0.01% means 0.01 mL of DMSO to 99.99 mL of water) in all groups and untreated controls. Cells were seeded in 96-well plates at a density of 5 × 10^3^ cells per well and incubated with compounds diluted in serial concentrations ranging from 0.29 µM to 150 µM. After 24, 48, and 72 h at 37 °C and 5% CO_2_, 10 µL of MTT (5 mg/mL) was added to each well and incubated for 4 h at 37 °C. The supernatant was then removed, and 100 µL/well of DMSO was added to dissolve any deposited formazan. The optical density was measured at 570 nm using a microplate reader (DR-200B-NM—Kasuaki, Tokyo, Japan).

### 3.10. Molecular Docking

The molecular docking protocol was developed using the orthorhombic crystallographic structure of the complex formed between the trypsin and the peptide Ptry9L; this structure was deposited in the Protein Data Bank (PDB) under the code 6EAT, with a resolution of 1.15 Å. Ptry9L is a nine-amino-acid residue cyclic peptide (Cis1-Thr2-Lys3-Ser4-Ile5-Pro6-Pro7-Gln8-Cys9-), which is derivate from the Black-eyed pea Trypsin and Chymotrypsin inhibitor (BTCI), which is a BBI family inhibitor isolated from *Vignia unguiculata* seed [61]. Ptry9L presented a disulfide bond between its L-Cis1 and L-Cys9 residue. Rigid–rigid docking simulations were executed using the AutoDockTools 4 package [70] with the AutoDock Vina algorithm [71,72]. This study was performed using the protocol set in our previous work [73]. For the grid box size, the dimensions of the search space were selected as 17.25 Å × 17.25 Å × 15.75 Å, using a grid spacing of 0.375 points.

In the redocking study of Ptry9L against trypsin, the value of the RMSD of the superposition between the conformation pose of the crystal structure of the Ptry9L and the unique docking pose generated by the AutoDock Vina was 0.69 Å, and the binding energy (score) was −10.70 kcal/mol. After this validation, the molecular docking studies of the complexes (**1–3**) and the ligands H_2_L^1^ and H_2_L^2^ were performed to determine the best compound and possible inhibitor candidate. The visualization of the results and interactions was obtained with Discovery Studio Visualizer V21.1.0.20298 software [74].

## 4. Conclusions

Three new square-planar Ni(II) complexes were synthesized by complexation between S-allyl-2-(2-hydroxyphenyl-ethylidene)dithiocarbazate or S-benzyl-2-(2-hydroxyphenyl-ethylidene) dithiocarbazate ligands and nickel chloride. It was possible to observe that the ligands were coordinated in a deprotonated way by O1, N1, and S2 atoms to the metal center. A neutral triphenylphosphine or pyridine molecule completed the coordination polyhedral. The data show good agreement between the single-crystal X-ray diffraction and physical chemistry and spectroscopic methods. The Hirshfeld surface results indicate that the greatest contributions to the crystal lattice were non-classical hydrogen bonds and π···π stacking interactions. The results of the anticancer studies confirmed that all compounds exhibited activity during in vitro screening. Most importantly, the three new compounds displayed lower IC_50_ values for the MCF7 breast cancer cell line, indicating greater potency in inhibiting cancerous cells compared with their non-malignant counterparts. These findings offer valuable insights into the potential anticancer effects of the new compounds and underscore the importance of further investigations into their biological activities. The compounds with the best energy of binding, and consequently with better inhibitory activity, were those able to perform interactions with the residues of the active site of the trypsin and mimic the Ptry9L profile; in this work, complex (**2**) showed the best activity as an inhibitor.

## Figures and Tables

**Table 1:** Selected bond lengths (Å) and angles (°) for the complexes (**1–3**), standard deviations in parentheses.

Bond Lengths (Å)			
	(1)	(2)	(3)
C1-O1	1.311(5)	1.313(3)	1.325(4)
C7-N1	1.330(5)	1.313(3)	1.328(4)
N1-N2	1.407(4)	1.414(3)	1.412(4)
N2-C9	1.277(5)	1.289(3)	1.295(4)
C9-S1	1.735(4)	1.729(3)	1.713(4)
C9-S2	1.743(4)	1.725(3)	1.756(4)
Ni1-O1	1.820(3)	1.836(17)	1.823(2)
Ni1-N1	1.896(3)	1.895(2)	1.851(3)
Ni1-S1	2.122(12)	2.127(8)	2.133(12)
Ni1-X	2.205(11)	2.200(8)	1.907(3)
**Bond Angles (°)**			
O1-Ni1-N1	94.11(12)	94.28(8)	96.12(12)
O1-Ni1-X	84.51(9)	86.19(6)	85.80(12)
X-Ni1-S1	93.23(4)	90.88(3)	89.73(10)
S1-Ni1-N1	88.77(10)	88.55(6)	88.62(10)
O1-Ni1-S1	173.95(10)	176.56(6)	174.12(9)
N1-Ni1-X	173.24(10)	176.92(6)	175.25(13)

X = P(1) for (**1**) and (**2**) and N(3) for (**3**).

**Table 2:** Angular strain and strain (cm^−1^) frequencies of the normal vibration modes selected for all compounds studied.

	H_2_L^1^	H_2_L^2^	(1)	(2)	(3)
ν(O-H)	3245	3418	-	-	-
ν(N-H)	3184	3179	-	-	-
ν(CSS)	1101	1057	982	939	984
ν(C=N)	1601	1601	1523	1561	1522
ν(C-O)	1219	1228	1239	1156	1239
ν(N-N)	936	987	1023	1014	1070
ν(C=N)_py_	-	-	-	-	1603
δ(Py)	-			-	690
Ν(C_PPh3_-P_PPh3_)	-	-	1070	1094	-
ν(C_PPh3_-C_PPh3_)			1435	1434	
ν(Ni-P_PPh3_)			692	693	

**Table 3:** Cytotoxic activities of the ligands H_2_L^1^ and H_2_L^2^ and its complexes (**1–3**) at 24 h, 48 h, and 72 h. The results are presented as the inhibition concentration that caused a 50% decrease in cell growth (IC_50_) against cells (values estimated by non-linear regression of data from a viability assessment).

IC_50_ (Inhibitory Concentration 50%) ± SD at 24 h					
Cell Line	H_2_L^1^	H_2_L^2^	(1)	(2)	(3)
**MCF-10A**	65.44 ± 0.034	86.10 ± 0.082	25.47 ± 0.031	30.24 ± 0.014	30.13 ± 0.018
**MCF-7**	88.85 ± 0.097	58.59 ± 0.018	12.82 ± 0.029	13.76 ± 0.025	14.37 ± 0.032
SI *	0.73	1.47	2.03	2.19	2.10
**IC_50_ (Inhibitory Concentration 50%) ± SD at 48 h**					
**MCF-10A**	62.86 ± 0.013	57.46 ± 0.032	28.43 ± 0.021	31.05 ± 0.010	20.11 ± 0.022
**MCF-7**	87.73 ± 0.042	49.47 ± 0.012	22.71 ± 0.016	12.53 ± 0.015	18.34 ± 0.012
SI *	0.71	1.16	1.25	2.48	1.10
**IC_50_ (Inhibitory Concentration 50%) ± SD at 72 h**					
**MCF-10A**	62.86 ± 0.025	57.46 ± 0.012	28.43 ± 0.011	31.05 ± 0.024	20.11 ± 0.012
**MCF-7**	28.49 ± 0.023	23.97 ± 0.015	18.07 ± 0.017	8.073 ± 0.011	14.38 ± 0.014
SI *	2.21	2.40	1.57	3.85	1.40

* The selectivity index (SI) is calculated as SI = IC_50_ in normal cells/IC_50_ in tumor cells [27].

**Table 4:** Docking energy of binding (kcal/mol) of the ligands H_2_L^1^ and H_2_L^2^ and their Ni(II) complexes (**1–3**), the number of hydrogen bond interactions (#HB) with the main residues of the active site of the trypsin, and the distance of the hydrogen bond (in Å) between the compound’s atoms and the NE2 atom of the Gly192 residue of the trypsin enzyme.

Molecule	Score	#HB	Distance
**(1)**	−6.60	1	2.99^S2^
**(2)**	−9.20	1	2.57^S2^
**(3)**	−7.80	0	-
**H_2_L^1^**	−5.40	0	-
**H_2_L^2^**	−5.80	0	-
**Ptry9L**	−10.70	9	2.48^Thr2:0^

## Data Availability

The datasets generated for this study can be found in the Supplementary Materials.

## Supplementary Materials

The following supporting information can be downloaded from https://www.mdpi.com/article/10.3390/molecules29122759/s1. Supplementary crystallographic data for the structures in this work were deposited at the Cambridge Crystallographic Data Centre, CCDC 2304153-2304155. Copies of the available material can be obtained free of charge by application to the Director, CCDC, 12 Union Road, Cambridge CH2 1EZ, UK (fax: +44 1223 336033; E-mail: deposit@ccdc.cam.ac.uk or http://www.ccdc.cam.ac.uk). Supplementary tables, figures, NMR, and IR spectra are as detailed in the text (PDF). Crystallographic data are in CIF files.
