# Supplementary material for: Structural Design, Anticancer Evaluation, and Molecular Docking of Newly Synthesized Ni(II) Complexes with ONS-Donor Dithiocarbazate Ligands
Source: Molecules. 2024 Jun 10;29(12):2759. doi: 10.3390/molecules29122759 (PMC11206525; doi:10.3390/molecules29122759)
Supplement: Supplementary file 1 [file molecules-29-02759-s001.zip › molecules-3036805-supplementary.pdf]

Supporting Information

Structural Design, Anticancer Evaluation and Molecular Docking of Newly Synthesized Ni(II) Complexes with *ONS*-Donor Dithiocarbazate Ligands

Summary

Figure S1. Projection view of (**1**) showing the hydrogen bonds present. III

Figure S2. Fingerprint plots for (**1**). IV

Figure S3. Fingerprint plots for (**2**). IV

Figure S4. Fingerprint plots for (**3**). V

Figure S5. FT-IR spectra of H_2L^1. V

Figure S6. FT-IR spectra of H_2L^2. VI

Figure S7. FT-IR spectra of (**1**). VI

Figure S8. FT-IR spectra of (**2**). VI

Figure S9. FT-IR spectra of (**3**). VII

Figure S10. UV-vis spectra of compound H_2L^1 and (**1**) in DMF. VII

Figure S11. UV-vis spectra of compound H_2L^2, (**2**) and (**3**) in DMF. VIII

Table S1. Results obtained in the electron spectroscopy analysis of all the compounds obtained with wavelength values in nm. IX

Figure S12. ^{1}H-NMR spectra of H_2L^1 IX

Figure S13. ^{13}C-NMR spectra of H_2L^1 IX

Figure S14. ^{1}H-NMR spectra of H_2L^2 X

Figure S15. ^{13}C-NMR spectra of H_2L^2 X

Figure S16. ^{1}H-NMR spectra of compound (**1**). XI

Figure S17. ^{13}C-NMR spectra of compound (**1**) XI

Figure S18. ^{1}H-NMR spectra of compound (**2**). XII

Figure S19. ^{13}C-NMR spectra of compound (**2**). XII

Figure S20. ^{1}H-NMR spectra of compound (**3**). XIII

Figure S21. ^{13}C-NMR spectra of compound (**3**) XIII

Figure S22. Evaluation of cytotoxic effects by MTT assay in 24 hours. DMSO at 0.01% did not affect the cell viability. The asterisk indicates that cell viability is significantly different from the respective DMSO control (*p < 0.5, **p < 0,001 Kruskal–Wallis followed by Dunn's comparison test). XIV

Figure S23. Evaluation of cytotoxic effects by MTT assay in 48 hours. DMSO at 0.01% did not affect the cell viability of cell lines. The asterisk indicates that cell viability is significantly different from the respective DMSO control (*p < 0.5, **p < 0,001 Kruskal–Wallis followed by Dunn's comparison test). XV
Table S2. X-ray diffraction data collection and refinement parameters for complexes (**1-3**)...XV

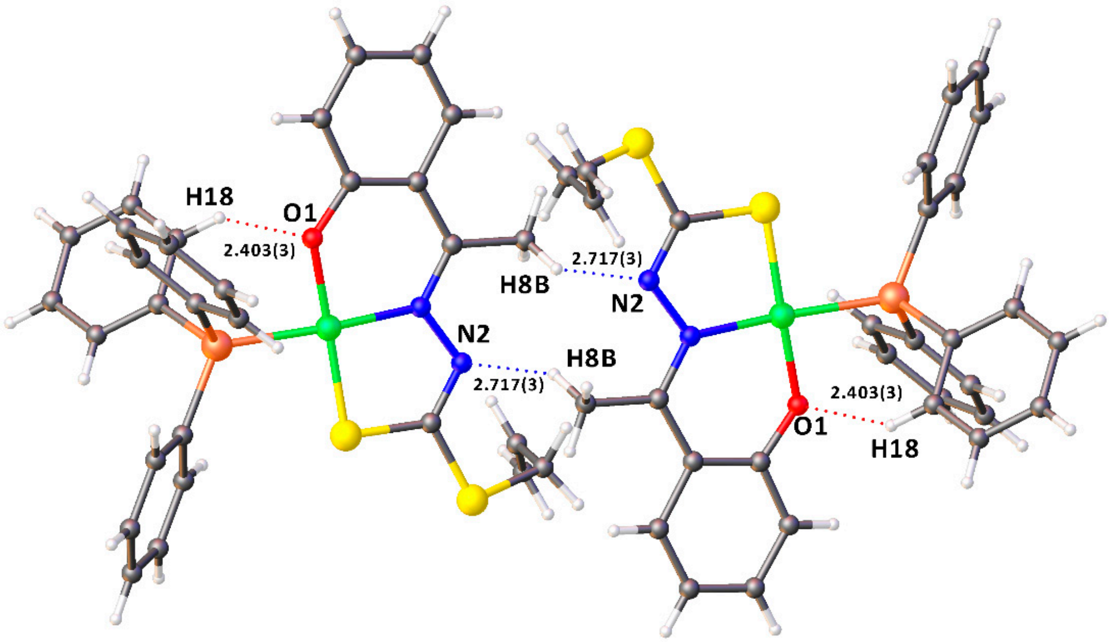

Figure S1. Projection view of (**1**) showing the hydrogen bonds present.

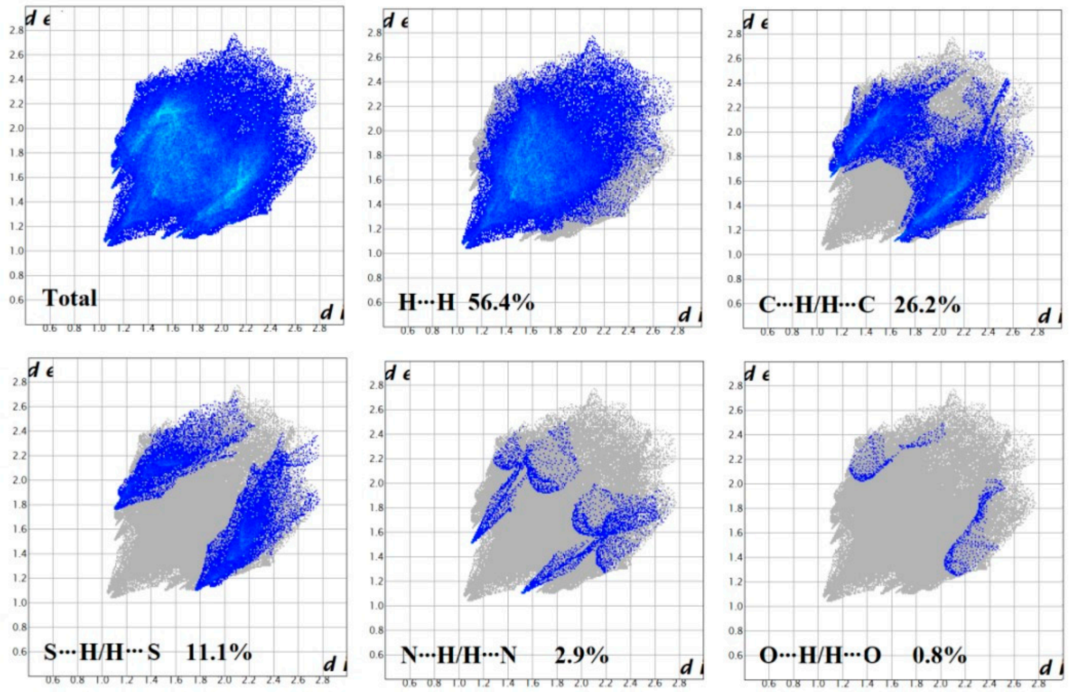

Figure S2. Fingerprint plots for (**1**).

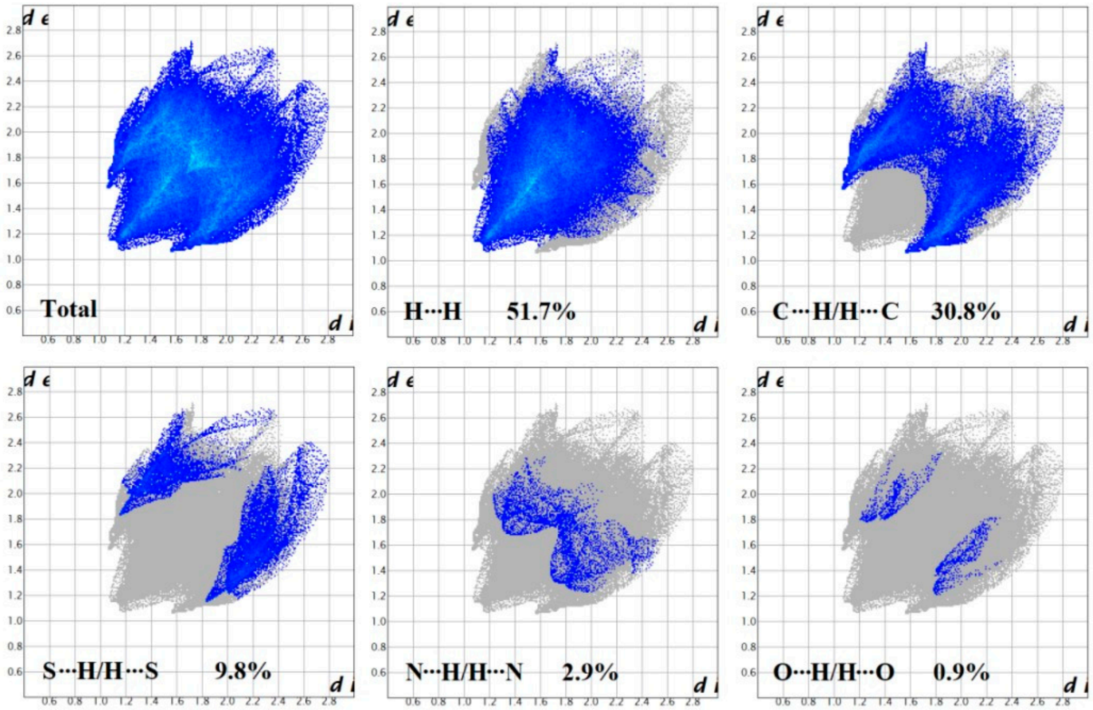

Figure S3. Fingerprint plots for (**2**).

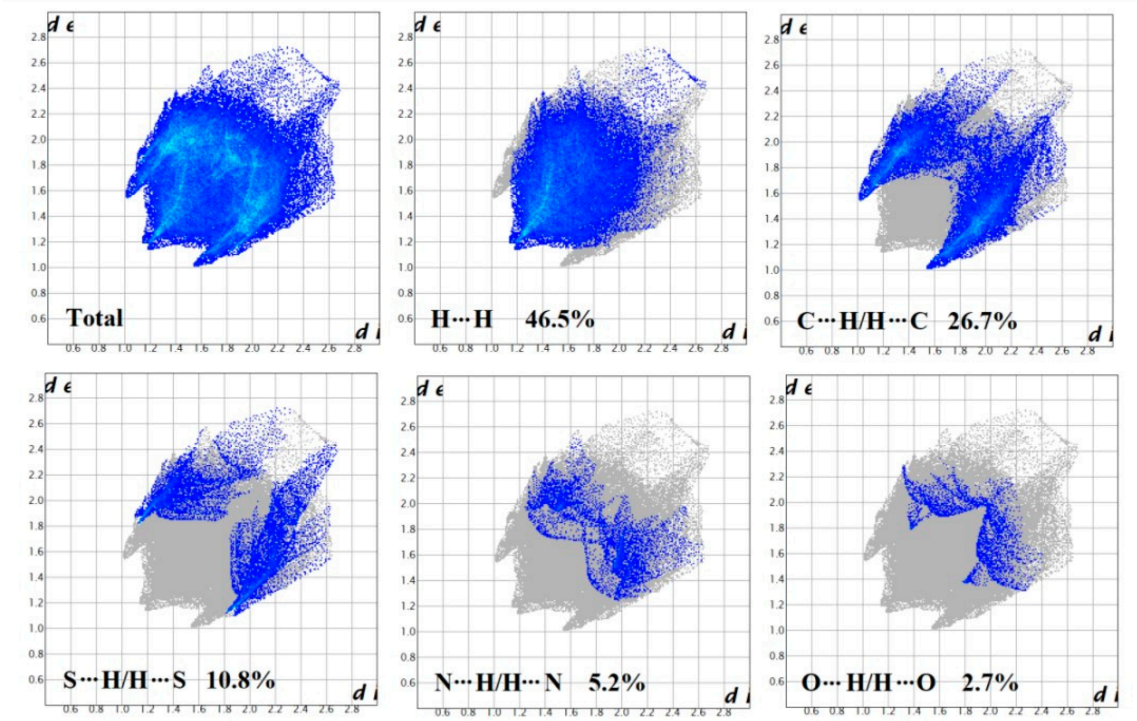

Figure S4. Fingerprint plots for (**3**).

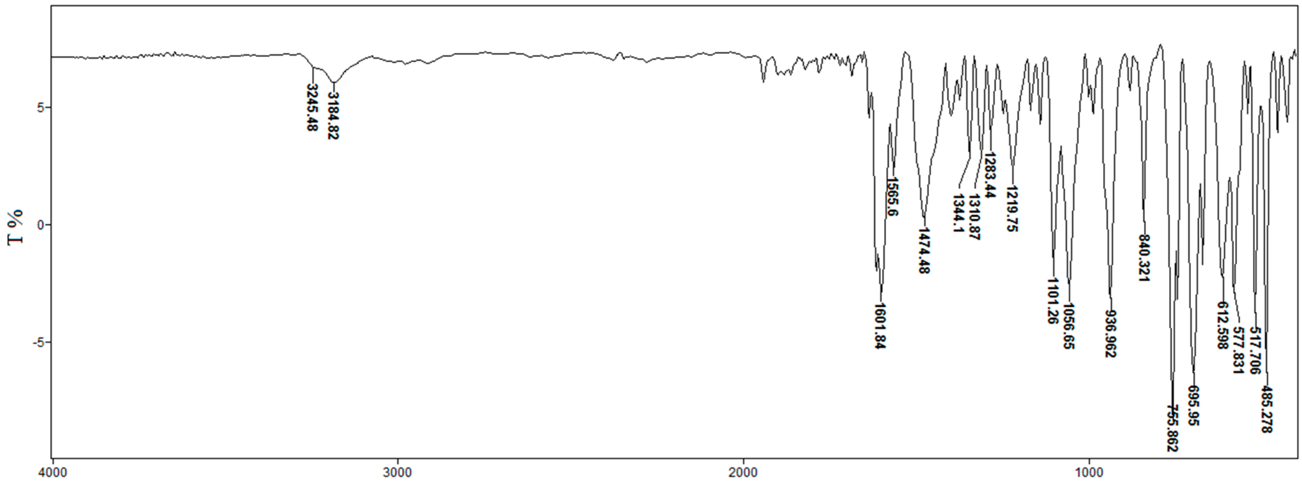

Figure S5. FT-IR spectra of H_2L^1.

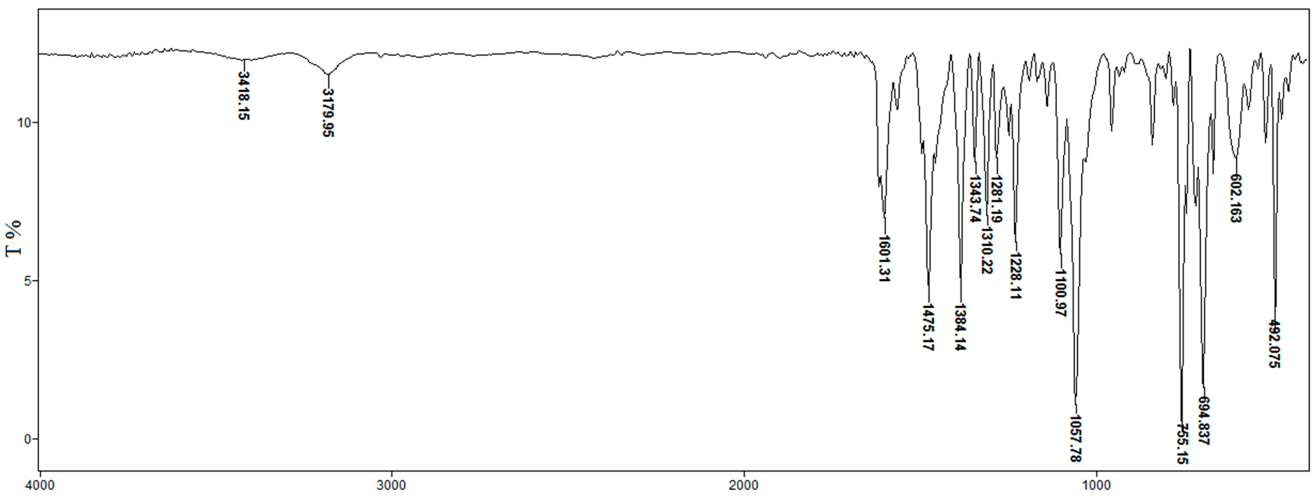

Figure S6. FT-IR spectra of H_2L^2.

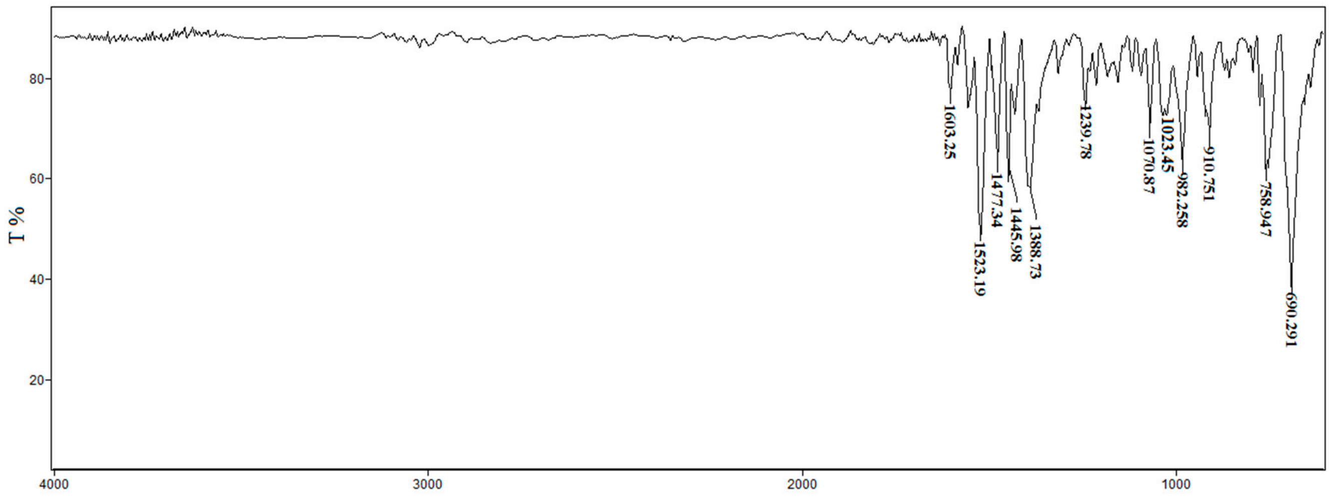

Figure S7. FT-IR spectra of (**1**).

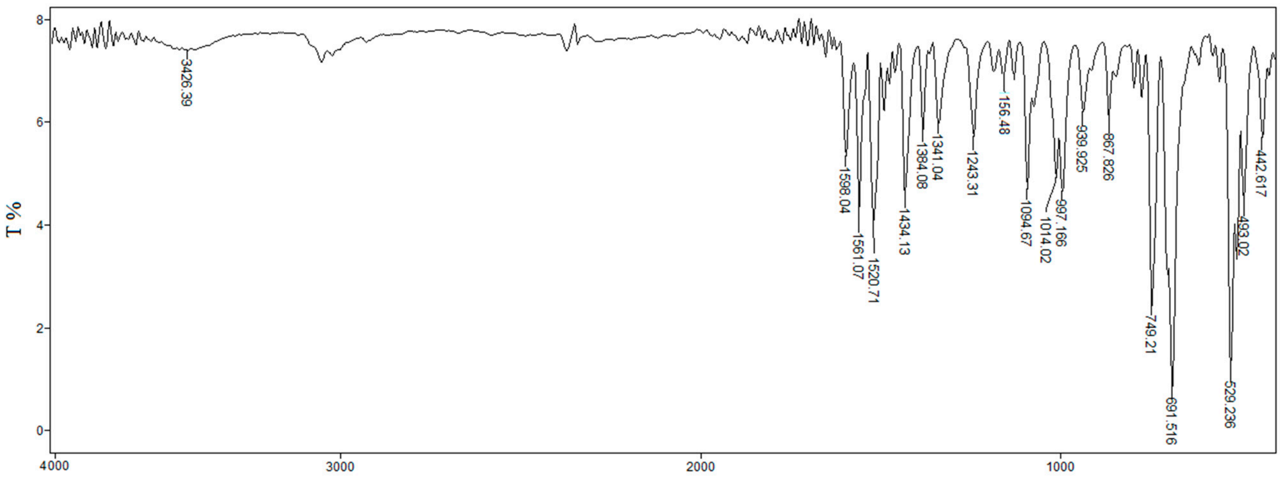

Figure S8. FT-IR spectra of (**2**).

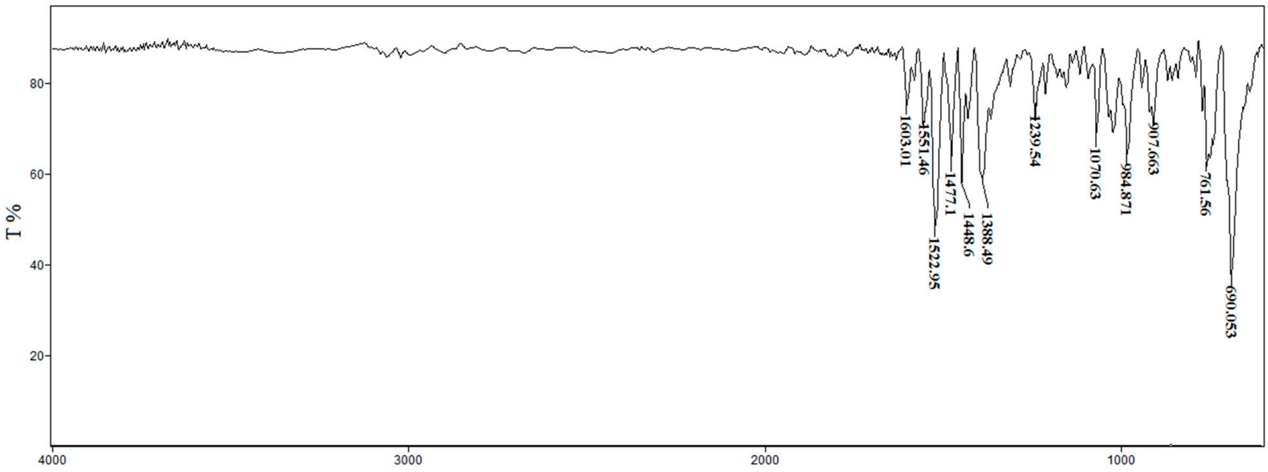

Figure S9. FT-IR spectra of (**3**).

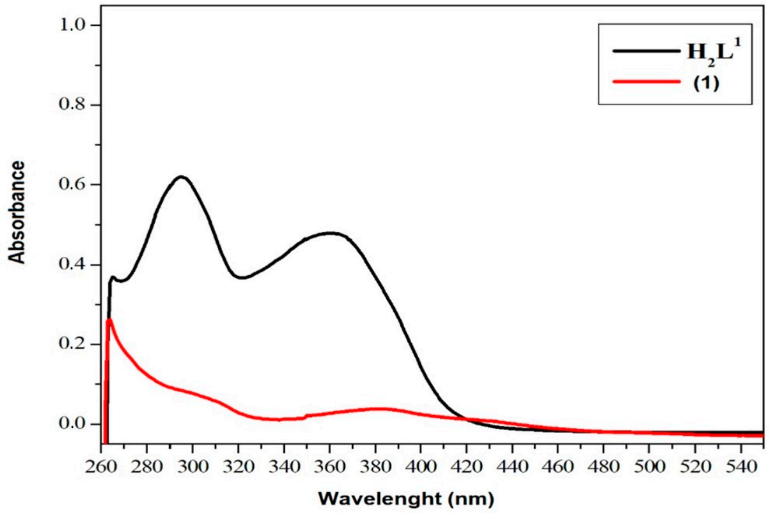

Figure S10. UV-vis spectra of compound H_2L^1 and (**1**) in DMF.

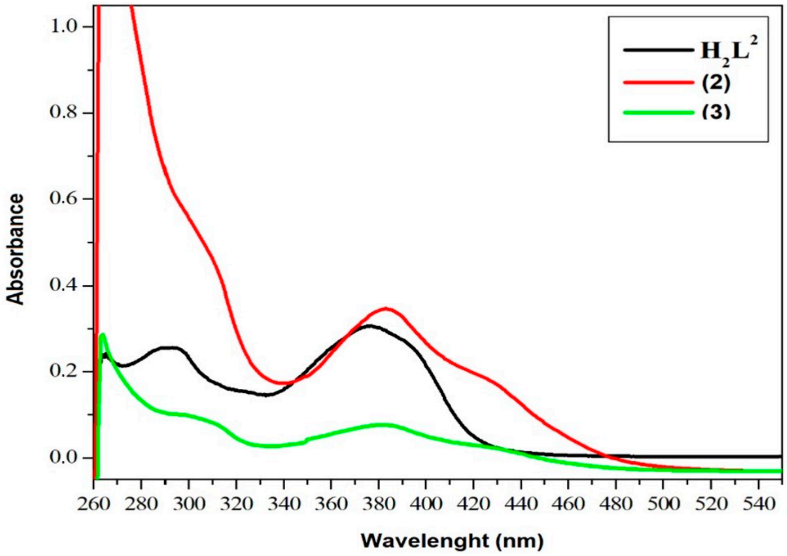

Figure S11. UV-vis spectra of compound H_2L^2, (**2**) and (**3**) in DMF.

Table S1. Results obtained in the electron spectroscopy analysis of all the compounds obtained with wavelength values in nm.

	π→ π*	log ε	n→π*	log ε	LMCT	log ε
**H_2L^1**	294	4.49	361	4.38	---	---
**H_2L^2**	292	4.11	376	4.18	---	---
**(1)**	304	3.56	379	3.32	428	---
**(2)**	308	4.38	383	4.24	428	3.95
**(3)**	305	3.66	381	3.60	428	3.09

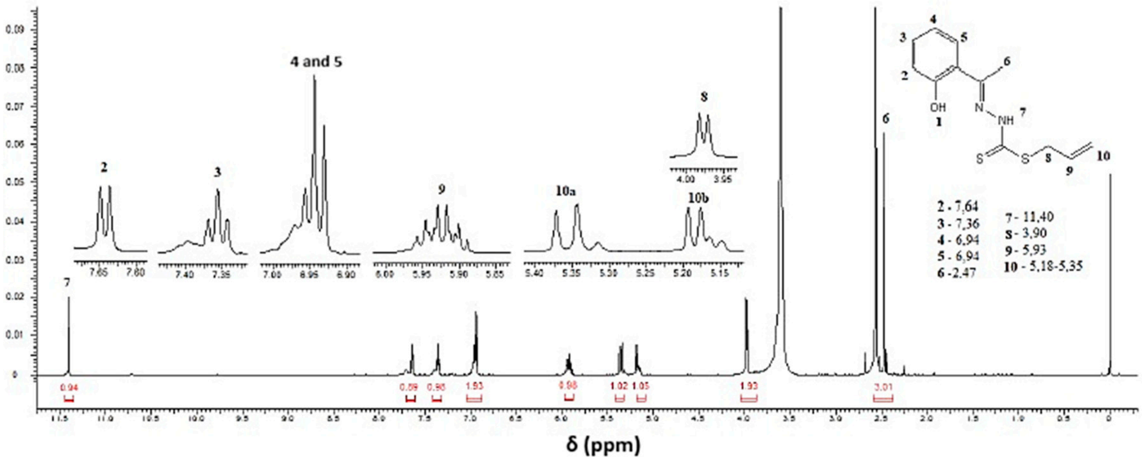

Figure S12. ^{1}H-NMR spectra of H_2L^1.

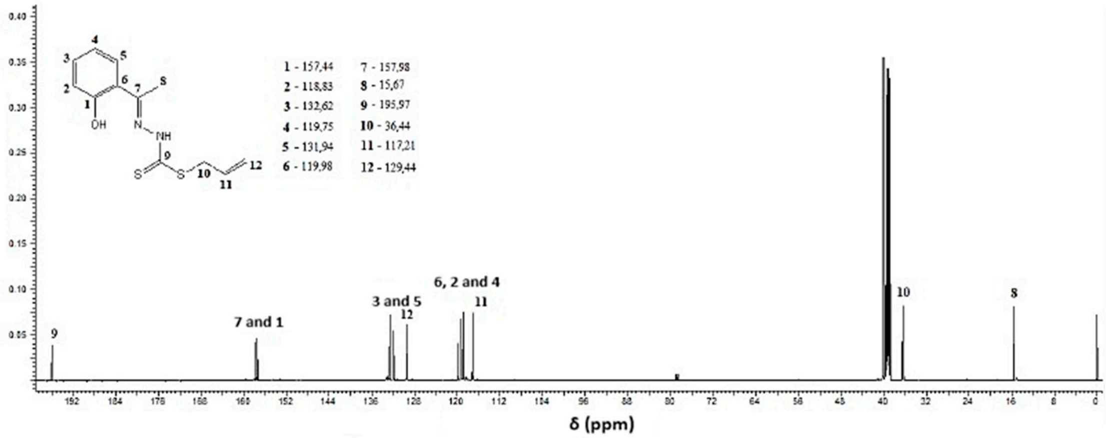

Figure S13. ^{13}C-NMR spectra of H_2L^1.

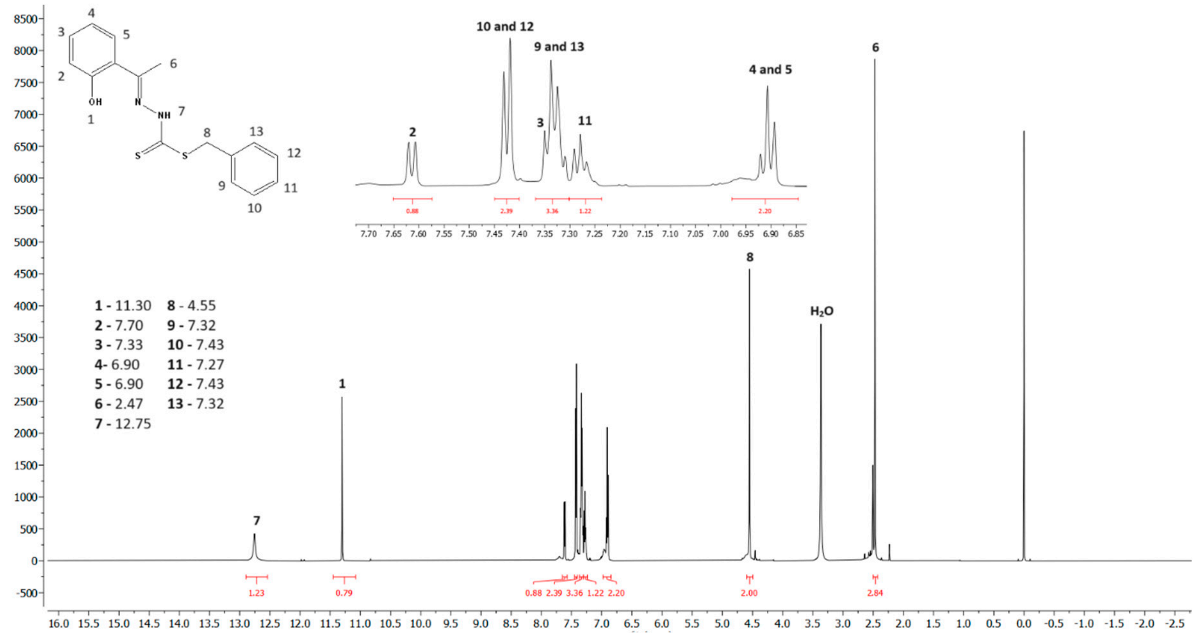

Figure S14. ^{1}H-NMR spectra of H_2L^2.

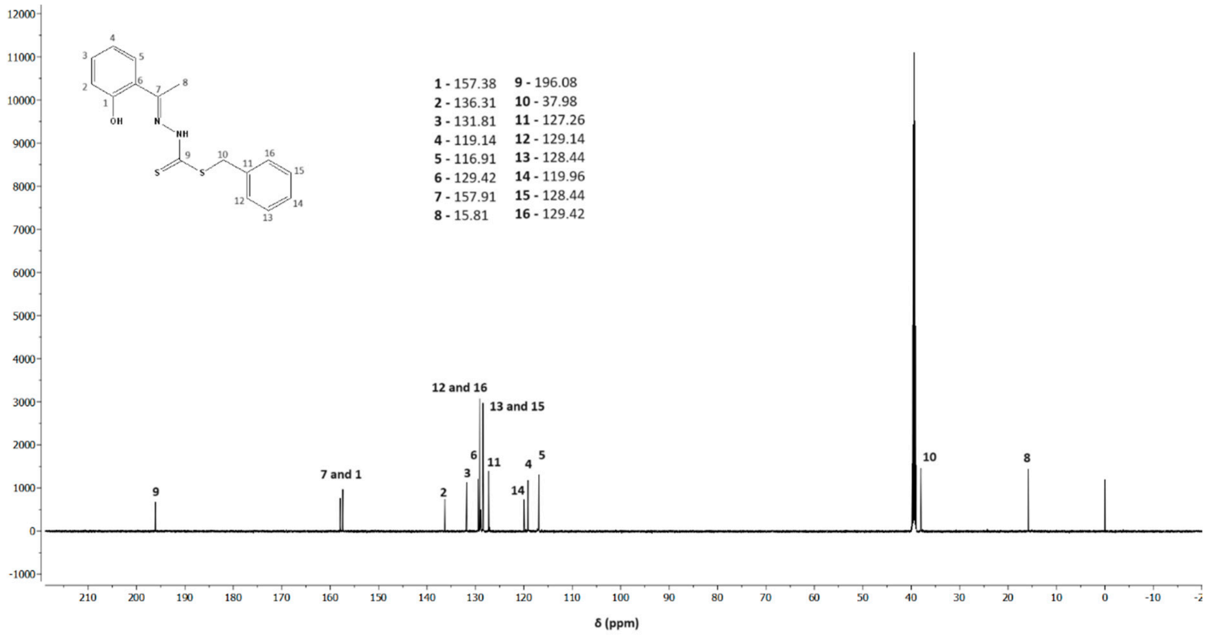

Figure S15. ^{13}C-NMR spectra of H_2L^2.

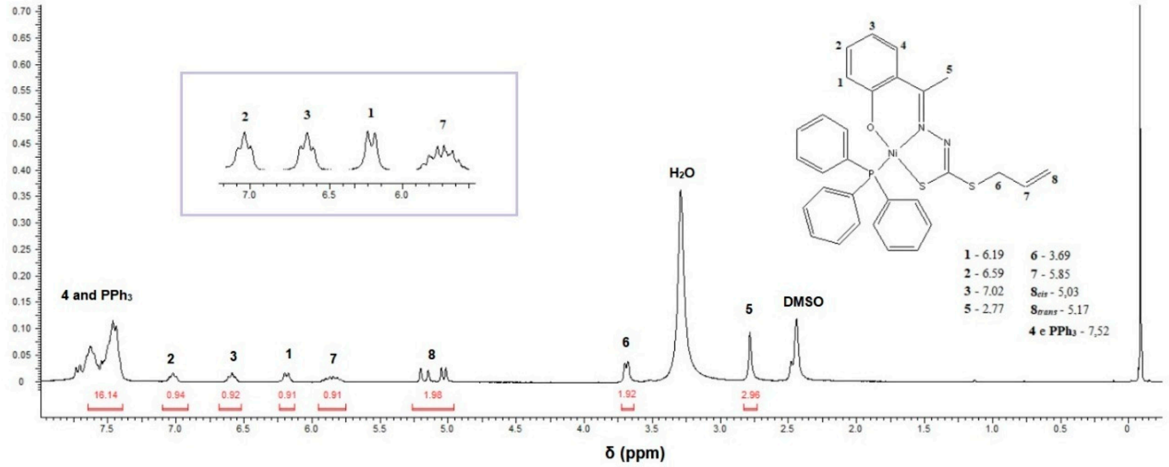

Figure S16. ¹H-NMR spectra of compound (**1**).

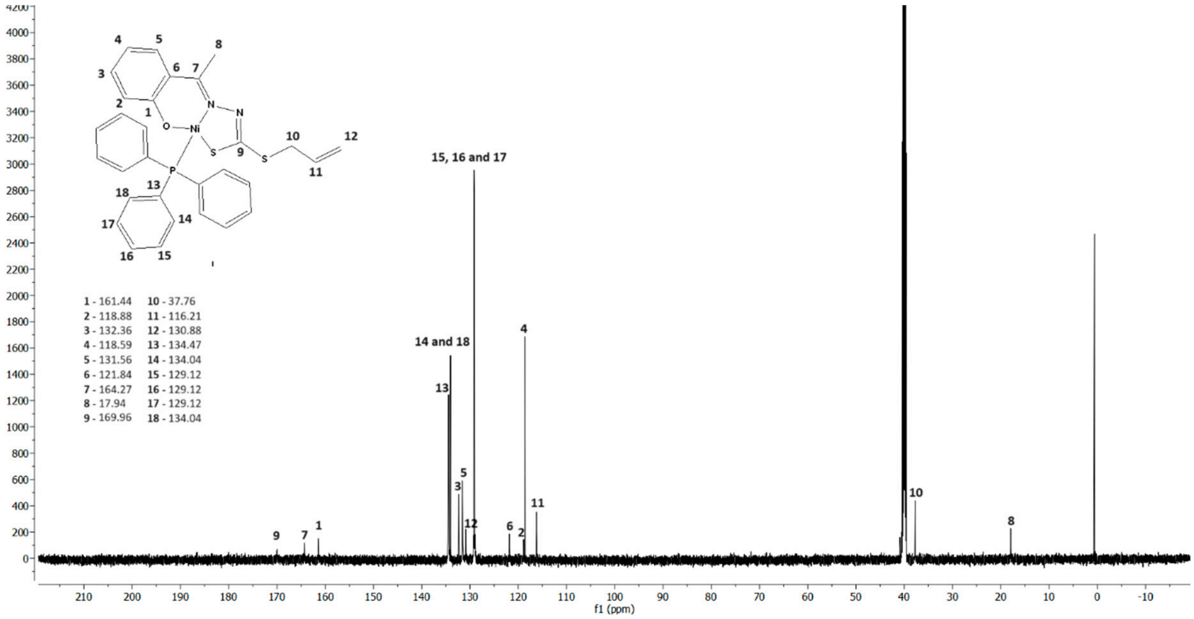

Figure S17. ¹³C-NMR spectra of compound (**1**).

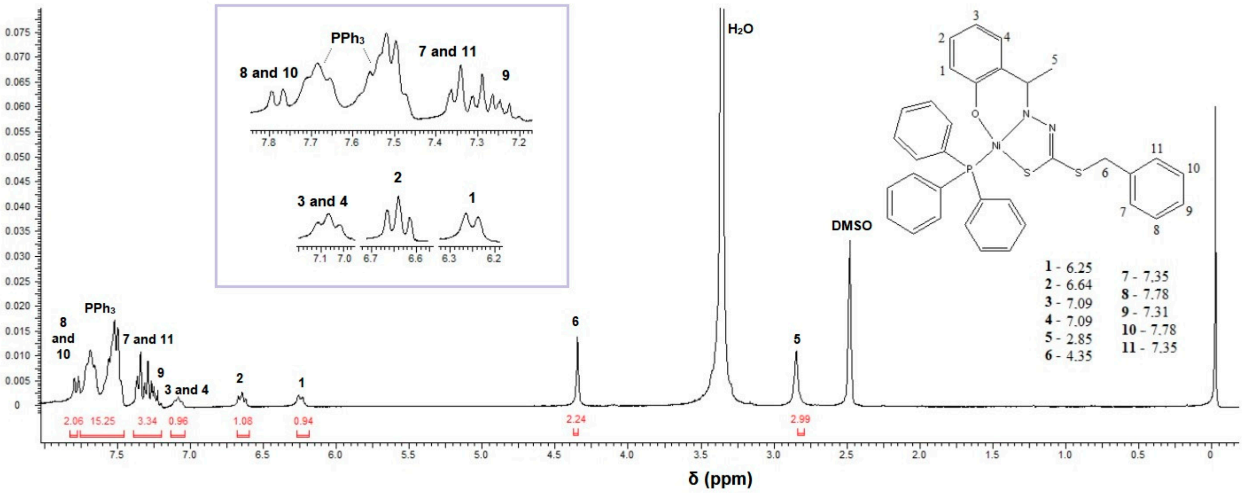

Figure S18. ^{1}H-NMR spectra of compound (**2**).

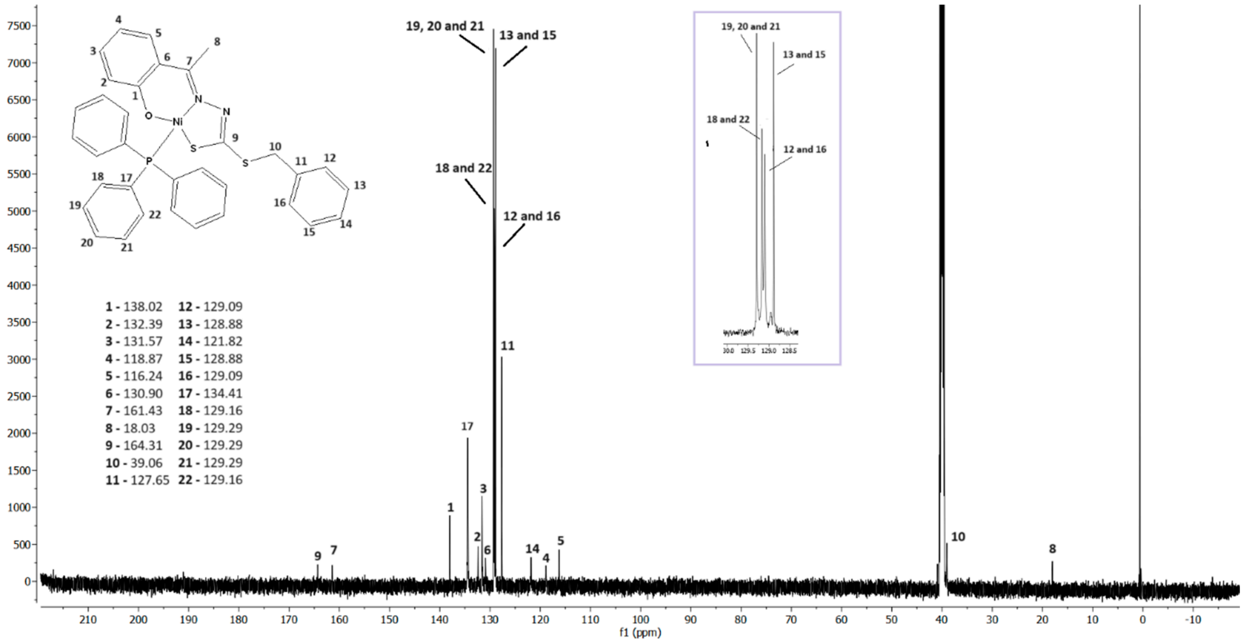

Figure S19. ^{13}C-NMR spectra of compound (**2**).

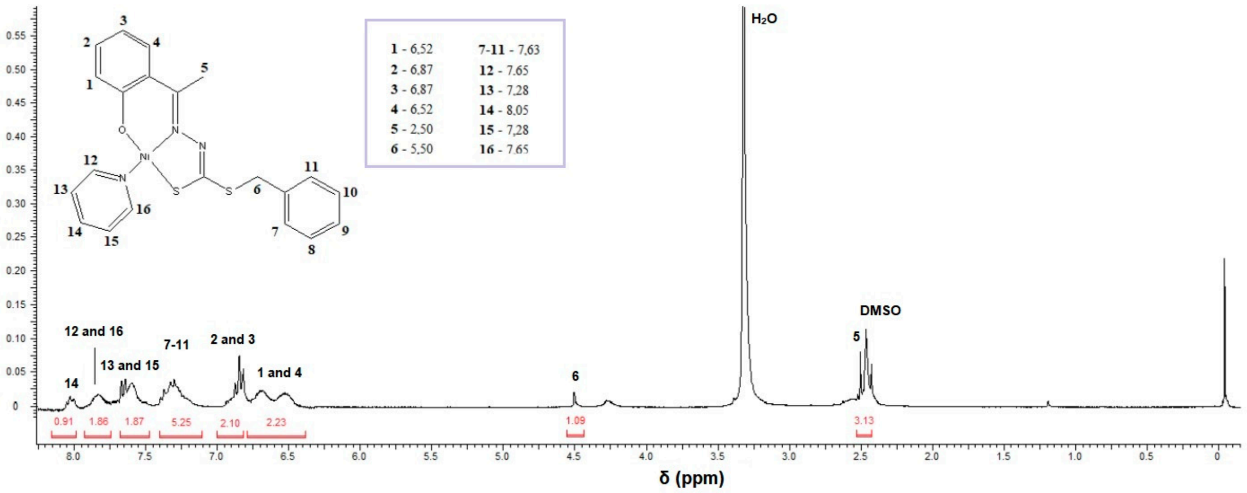

Figure S20. ^{1}H-NMR spectra of compound (**3**).

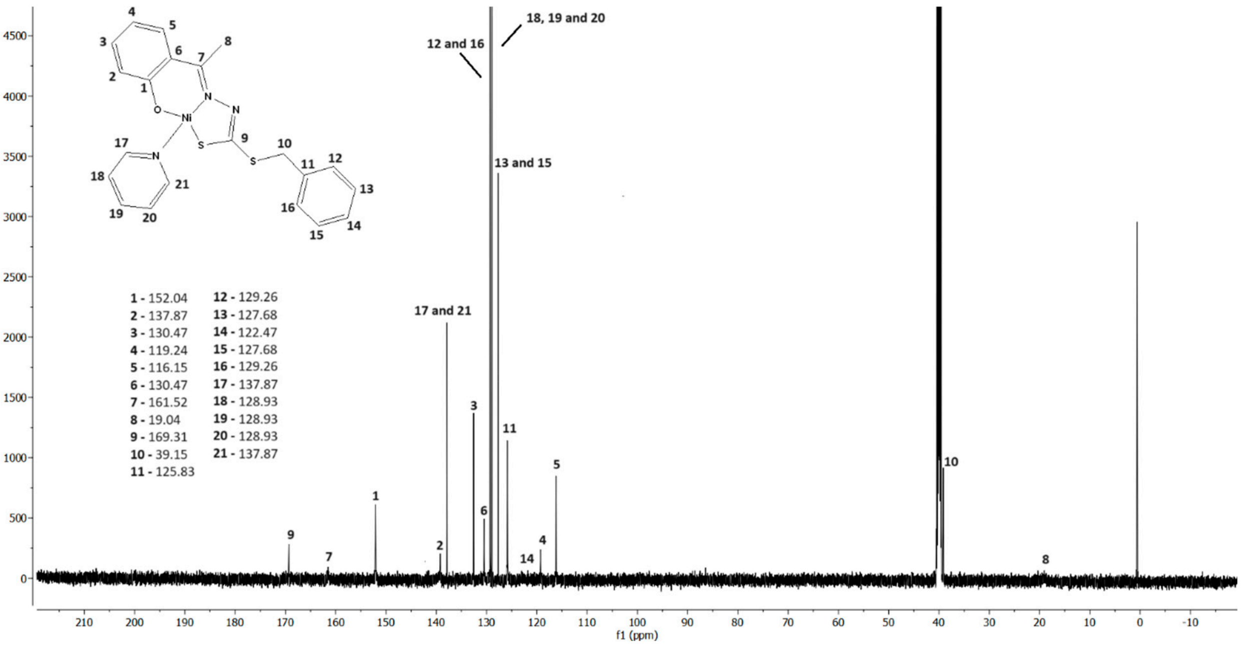

Figure S21. ^{13}C-NMR spectra of compound (**3**).

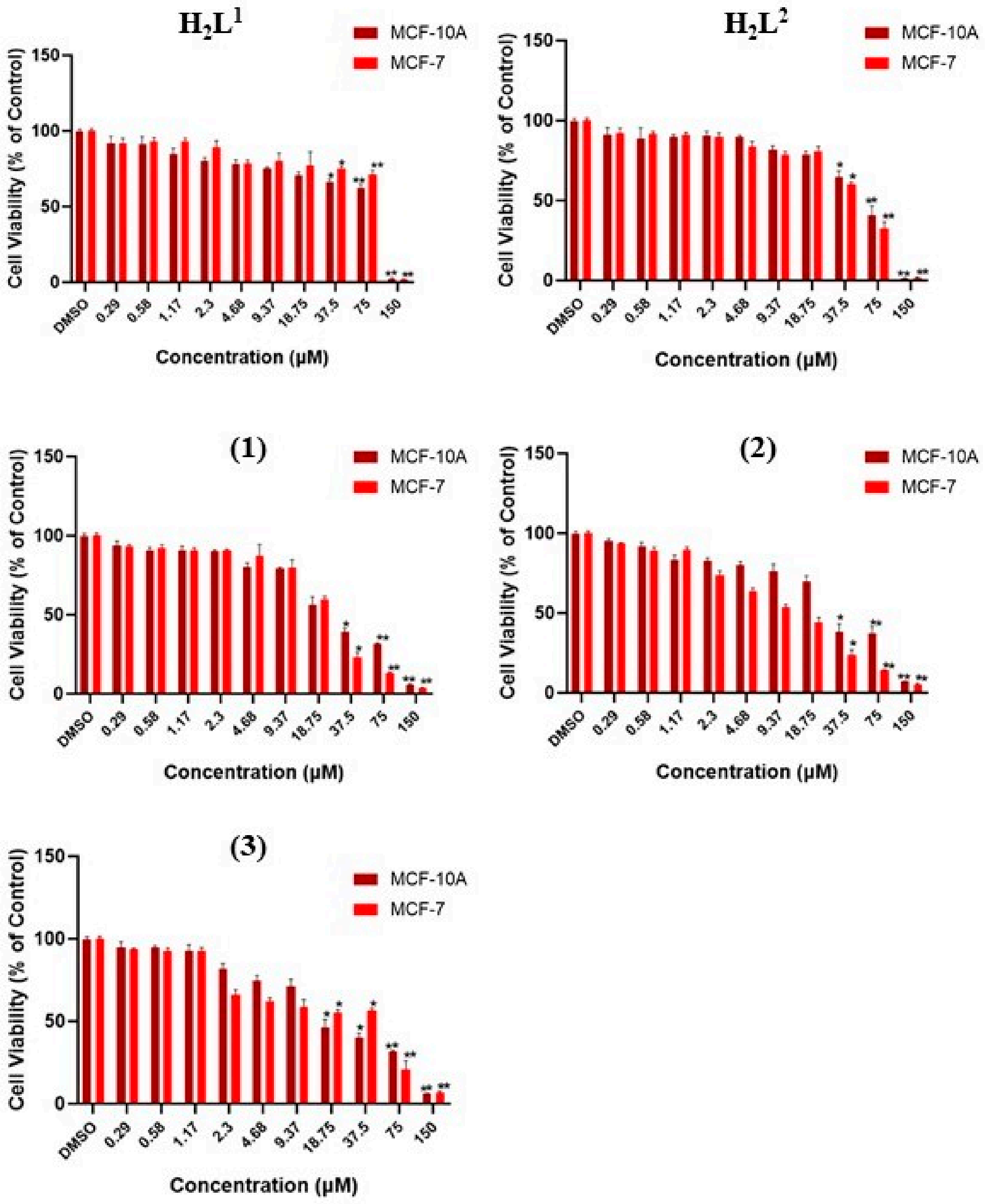

Figure S22. Evaluation of cytotoxic effects by MTT assay in 24 hours. DMSO at 0.01% did not affect the cell viability. The asterisk indicates that cell viability is significantly different from the respective DMSO control (*p < 0.5, **p < 0,001 Kruskal–Wallis followed by Dunn's comparison test).

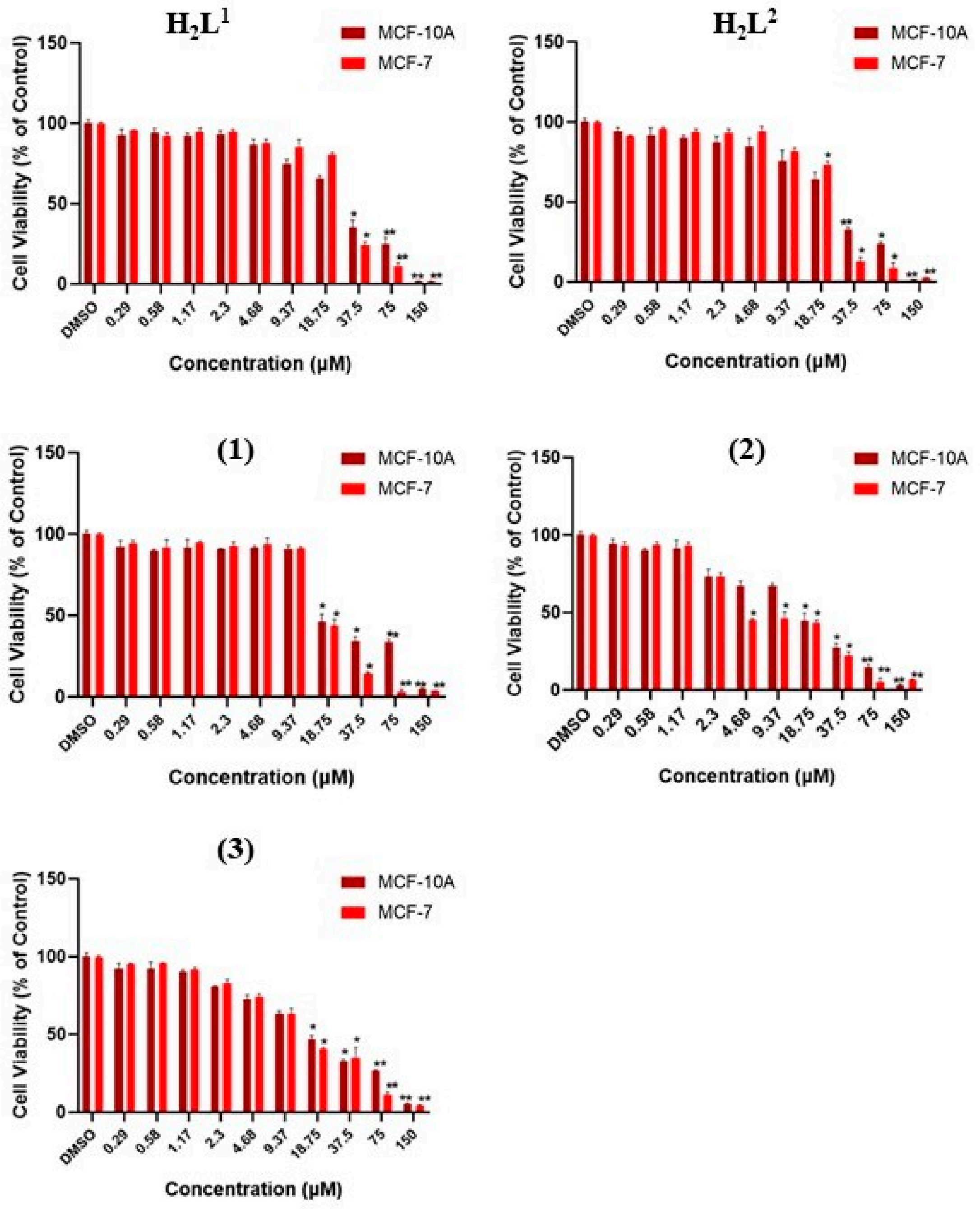

Figure S23. Evaluation of cytotoxic effects by MTT assay in 48 hours. DMSO at 0.01% did not affect the cell viability of cell lines. The asterisk indicates that cell viability is significantly different from the respective DMSO control (*p < 0.5, **p < 0,001 Kruskal–Wallis followed by Dunn's comparison test).

Table S2. X-ray diffraction data collection and refinement parameters for complexes (**1-3**).

	**(1)**	**(2)**	**(3)**
Empirical formula	$C_{30}H_{27}NiN_2OPS_2$	$C_{34}H_{29}NiN_2OPS_2$	$C_{21}H_{19}NiN_3OS_2$
Formula weight	585.33	635.39	452.22
Crystal System	triclinic	triclinic	triclinic
Space group	P-1	P-1	P-1

a (Å)	9.244(12)	9.057(17)	9.040(2)
b (Å)	9.630(13)	13.744(3)	10.768(3)
c (Å)	16.240(2)	14.331(3)	11.781(3)
α (°)	87.261(3)	115.278(3)	71.462(5)
β (°)	80.332(3)	94.584(3)	73.142(5)
γ (°)	82.033(3)	107.284(3)	68.263(5)
V ($Å^3$)	1410.9(3)	1495.5(5)	990.5(5)
Z	2	2	2
Density ($Mg{\cdot}cm^{-3}$)	1.378	1.411	1.516
Index ranges	$-11 \leq h \leq 11$	$-11 \leq h \leq 11$	$-10 \leq h \leq 10$
	$-11 \leq k \leq 11$	$-17 \leq k \leq 17$	$-13 \leq k \leq 13$
	$-19 \leq l \leq 19$	$-17 \leq l \leq 17$	$-14 \leq l \leq 14$
Absorption coefficient (mm^{-1})	0.918	0873	1.207
Absorption correction	multi-scan	multi-scan	multi-scan
Reflections collected	33021	26476	23214
Independent reflections/Rint	5190/0.1092	6110/0.0621	3666/0.1059
Data/restraints/param.	5190/0/335	6110/0/371	3666/0/255
R_1/wR_2 [I > 2s(I)]	0.050/0.103	0.039/ 0.083	0.045/ 0.084
Goodness-of-fit on F^2	1.017	1.059	0.999
Largest diff. peak and hole ($eÅ^{-3}$)	0.279 and -0.270	0.281 and -0.327	0.427 and -0.276
CCDC			
